# A dual enhancer-attenuator element ensures transient *Cdx2* expression during mouse posterior body formation

**DOI:** 10.1016/j.devcel.2025.06.006

**Published:** 2025-09-22

**Authors:** Irène Amblard, Damir Baranasic, Sheila Q. Xie, Benjamin Moyon, Michelle Percharde, Boris Lenhard, Vicki Metzis

**Affiliations:** 1 Institute of Clinical Sciences, Faculty of Medicine, Imperial College London, London W12 0HS, UK; 2 Medical Research Council (MRC) Laboratory of Medical Sciences (LMS), London W12 0HS, UK; 3 Division of Electronics, Ruder Boskovic Institute, Bijenicka cesta 54, 10000 Zagreb, Croatia

**Keywords:** enhancer, silencer, attenuator, motif composition, WNT signaling, retinoic acid nuclear receptor, CDX, spinal cord development, directed differentiation of mouse embryonic stem cells

## Abstract

During development, cells express precise gene expression programs to assemble the trunk of the body plan. Appropriate control over the duration of the transcription factor *Cdx2* is critical to achieve this outcome, yet how cells control the onset, maintenance, or termination of *Cdx2* has remained unclear. Here, we delineate the *cis*-regulatory logic orchestrating dynamic *Cdx2* expression in mouse caudal epiblast progenitors and their derivatives. Combining CRISPR-mediated deletion of regulatory elements with *in vitro* models and *in vivo* validation, we demonstrate that distinct enhancers and a repressive element embedded at the *Cdx2* locus act sequentially to drive transient *Cdx2* expression. We pinpoint an “attenuator”: a minimal region relying on a nuclear receptor to extinguish *Cdx2*. Changing this single motif converts the attenuator to an enhancer with the opposite regulatory behavior. Our findings establish a dual *cis*-regulatory logic ensuring precise spatiotemporal control over gene expression for vertebrate body patterning.

## Highlights

• Transient *Cdx2* expression depends on a dual enhancer-attenuator element
• A single nuclear receptor motif substitution converts an attenuator into an enhancer
• The *Cdx2* attenuator is required *in vivo* for mouse posterior body formation

## Graphical abstract

**Figure:**
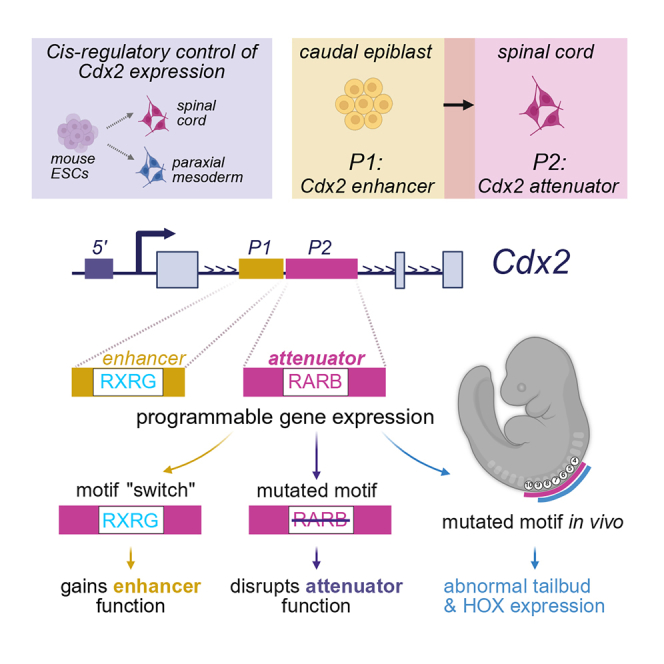

## Teaser

Amblard et al. dissect the function of *cis*-regulatory elements regulating transient *Cdx2* expression during mouse caudal body formation. They highlight the requirement of an attenuator, a transiently repressive element, which can be converted into an enhancer through a single nuclear receptor motif substitution.

## Introduction

In the mammalian body, a striking array of cell types emerges during development in response to extrinsic cues. This immense diversity results from the activity of gene regulatory networks that define cell identity.^1^ Yet, how cells interpret extrinsic signaling in a context-dependent manner to ensure the generation of different cell types remains a major open question.

CDX (Caudal Type Homeobox; CDX1, 2, and 4) transcription factors (TFs) play a central role in the development of the caudal part of the body plan.^2^^,^^3^^,^^4^^,^^5^ Removal of these partially redundant factors^6^^,^^7^ results in the loss of most post-occipital tissues, in part due to their regulation of homeobox (*Hox*) genes.^3^^,^^4^^,^^5^^,^^8^^,^^9^^,^^10^ Reduced or prolonged expression of CDX factors respectively truncates or expands the territory that forms the spinal cord (SC), at the expense of hindbrain fates, in multiple species.^6^^,^^11^^,^^12^^,^^13^^,^^14^^,^^15^ Unlike *Cdx1* and *Cdx4*, however, genetic removal of *Cdx2* alone demonstrates its indispensable role in posterior body formation.^3^^,^^6^^,^^7^^,^^8^^,^^16^^,^^17^

The expression of *Cdx2* is detected during gastrulation in the mouse caudal epiblast (CEpi).^18^^,^^19^^,^^20^ This region of the embryo harbors neuromesodermal progenitors (NMPs), a progenitor source that contributes to the developing SC and somites.^21^^,^^22^^,^^23^ Although *Cdx2* is detected in NMPs^24^^,^^25^^,^^26^ and is later maintained in the hindgut,^18^^,^^26^ it is only transiently expressed in tissues derived from NMPs, such as the SC and somites.^3^^,^^9^^,^^26^^,^^27^ WNT and fibroblast growth factor (FGF) signaling promote caudal embryo development and the expression of *Cdx2*.^12^^,^^13^^,^^28^^,^^29^ Similar regulation is observed *in vitro* using the directed differentiation of mouse or human embryonic stem cells (ESCs).^30^^,^^31^^,^^32^^,^^33^^,^^34^ In addition, retinoic acid (RA) signaling is a critical determinant of posterior body formation and differentiation^35^^,^^36^ and restricts the expression of *Cdx2* in the SC *in vivo*^27^ and *in vitro*.^24^^,^^37^ The transition from a CEpi to an SC progenitor coincides with a switch from FGF to RA signaling.^35^^,^^36^ How cells coordinate the duration of *Cdx2* expression in response to extrinsic cues remains unresolved.

Extrinsic cues are interpreted by *cis*-regulatory elements (CREs) to control gene expression. CREs encompass a broad range of elements that include promoters, enhancers,^38^ silencers,^39^ and insulators.^40^ Recent findings suggest that enhancers may function in concert with additional elements, such as tethers^41^ and, more recently, facilitators,^42^ yet how common such elements are in the genome remains unclear. Although many classes of CREs exist, the ability to predict the cellular context and function of individual CREs remains challenging.^43^ Several *Cdx2* enhancers have been identified that partially recapitulate the trophectoderm, CEpi, or intestinal expression pattern of *Cdx2 in vivo*.^19^^,^^44^^,^^45^^,^^46^^,^^47^^,^^48^ These studies suggest that a subset of *Cdx2* CREs play tissue-specific roles. Although long-range interactions can play a vital role in regulating developmental genes,^49^^,^^50^^,^^51^^,^^52^ the expression of *Cdx2* during posterior body formation appears to be regulated by elements located within the *Cdx2* locus. In particular, an 11 kb region flanking *Cdx2* recapitulates the caudal tailbud expression pattern of *Cdx2* between embryonic day (E)7.5 and E10.5.^53^ Strikingly, several CREs located within this region demonstrate enhancer activity in transgenic reporter embryos, yet, as individual elements, they do not recapitulate the full expression pattern of *Cdx2*.^46^^,^^53^ How multiple CREs within their native genomic context facilitate *Cdx2* initiation, maintenance, or termination remains unclear.

In this study, we investigate the regulatory mechanisms responsible for controlling the duration of *Cdx2* during the formation of posterior body derivatives: SC and paraxial presomitic mesoderm (PSM) progenitors. To dissect the molecular mechanisms that control *Cdx2*, without compromising axial elongation or trophectoderm specification,^54^ we used genome engineering approaches, combined with the directed differentiation of pluripotent ESCs, to model posterior body formation *in vitro*. Using this strategy, we provide evidence that the transient expression of *Cdx2* in cells relies on the sequential usage of CREs that perform discrete, nonredundant functions during development. We identify the location of a CRE that limits the duration of *Cdx2*. We demonstrate that this repressive element can be converted into an enhancer through a single RA nuclear receptor motif substitution. Furthermore, we validate that its function is critically dependent on the presence of the motif *in vitro* and during caudal body development *in vivo*. Taken together, we provide evidence that the composition and number of RA nuclear receptor motifs dictate regulatory element function and underpin the context-specific regulation of *Cdx2* during posterior body development.

## Results

### *Cdx2* CREs display transient accessibility during SC development

**Figure 1:**
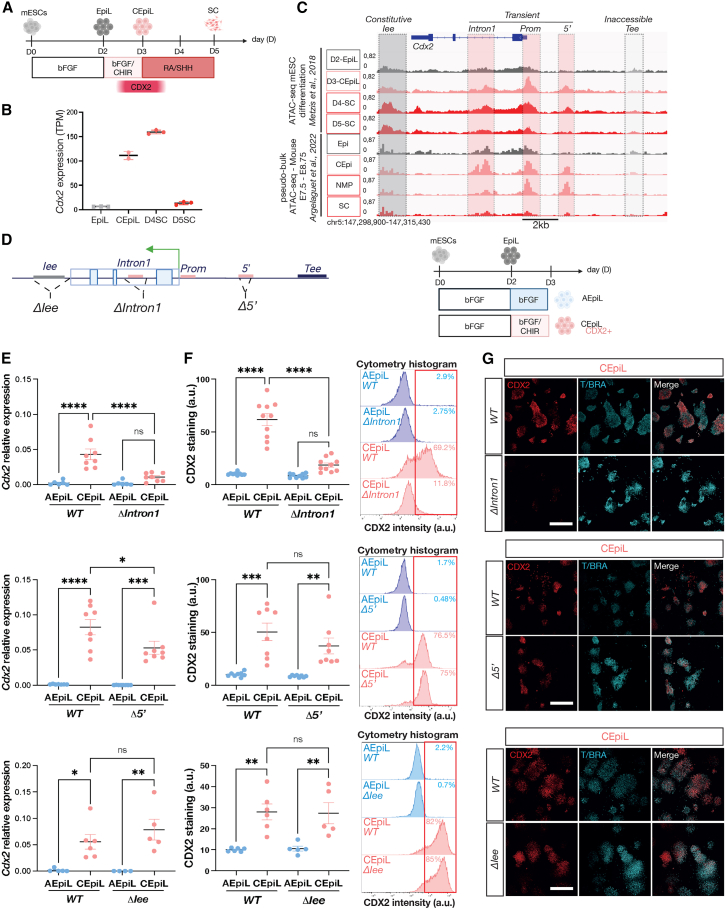
An intronic enhancer is indispensable for the onset of *Cdx2* in CEpi conditions (A) Simplified schematic of the 5-day SC progenitor differentiation from mESCs. (B) Reprocessed mRNA-seq data demonstrate *Cdx2* expression is transiently induced.^31^ (C) Bulk ATAC-seq signal at the *Cdx2* locus between days 2 and 5^55^ and single-nucleus pseudobulk ATAC-seq signals at the *Cdx2* locus in indicated *in vivo* cell types.^56^ Pink shading highlights the promoter (*Prom*), *Intron1*, and *5′* elements. The intestinal (*Iee*) and trophectoderm enhancer (*Tee*) are highlighted in dark and light gray shading, respectively. (D) Schematic of the *Cdx2* regulatory elements targeted to remove the *Iee* (*ΔIee*), first intron (*ΔIntron1*) or *5′* element (*Δ5′*) and conditions used to induce *Cdx2 in vitro* in CEpiL cells versus AEpiL cells used as a control. (E) Relative expression (RT-qPCR) for *Cdx2* in AEpiL (blue) versus CEpiL (pink) conditions collected from *ΔIntron1*, Δ*5′*, and *ΔIee* showing *Cdx2* is not induced in *ΔIntron1* cells, while *Δ5′* cells display a slight decrease. (F) Flow cytometry quantification of CDX2 levels in AEpiL (blue) versus CEpiL (pink) conditions collected from *ΔIntron1*, Δ*5′*, and *ΔIee* cells showing CDX2 is not induced in *ΔIntron1* cells and representative flow cytometry histograms for CDX2 indicating the proportion of CDX2-positive cells. (G) IF demonstrates that *ΔIntron1* cells maintain expression of TBXT (T/BRA) in CEpiL conditions despite loss of CDX2. Scale bar, 500 μm. *n* = 3. (A) and (D) created with BioRender.com. Data are represented as mean ± SEM. CEpiL, caudal epiblast-like; EpiL, epiblast-like; mESCs, mouse embryonic stem cells; SC, spinal cord; TPM, transcripts per million. ∗*p* value < 0.05, ∗∗*p* value < 0.01, ∗∗∗*p* value < 0.001, ∗∗∗∗*p* value < 0.0001.

To identify putative CREs regulating *Cdx2* expression during posterior body formation, we examined the chromatin accessibility landscape inferred from assay for transposase-accessible chromatin with sequencing (ATAC-seq) experiments using two different approaches. We complemented an *in vitro* time course of bulk ATAC-seq data from mouse ESCs differentiated into SC progenitors, which transiently express *Cdx2*^55^ (Figures 1A–1C), with pseudobulk ATAC-seq profiles obtained from the corresponding cell types present *in vivo*, extracted from 10× multiome single-nucleus (sn)ATAC-seq performed on E7.5–E8.75 whole embryos^56^ (Figure 1C). The transient expression of *Cdx2 in vitro* corresponds to CEpi-like (CEpiL) cells, which, upon differentiation to SC, lose *Cdx2*^31^^,^^55^^,^^57^ (Figures 1A and 1B). As genome-wide changes in chromatin accessibility take place in CEpiL versus SC progenitors,^55^^,^^57^^,^^58^ we hypothesized that changes in the availability of CREs may contribute to the regulation of *Cdx2*.

Consistent with this, known *Cdx2* CREs^19^^,^^44^^,^^45^^,^^46^^,^^47^^,^^53^^,^^59^ (Figure S1A) exhibited distinct patterns of chromatin accessibility (Figures 1C and S1B). In particular, a set of three CREs were transiently accessible *in vitro* when comparing CEpiL (*Cdx2* positive; Figures 1B, 1C, and S1B) to SC progenitors (*Cdx2* lacking; Figures 1B, 1C, and S1B). These corresponded to the *Cdx2* promoter, an *Intron1* CRE,^19^^,^^53^ and a CRE located upstream of the transcriptional start site of *Cdx2*,^53^ termed *5′* (Figure 1C, “transient,” boxed in pink). Similarly, *in vivo*, these regions are accessible in the CEpi (Figure 1C) but appear inaccessible in SC progenitors (Figure 1C). Both the *Intron1* and *5′* CRE exhibit enhancer activity in posterior tissues, although their onset and specificity differ from *Cdx2*.^53^ By contrast, CREs that regulate *Cdx2* in other tissues such as the intestine (*Iee*)^46^^,^^47^ or trophectoderm enhancer (*Tee*)^44^^,^^45^ appear either continuously accessible (“constitutive,” Figure 1C; box shaded in gray) or largely lacking accessibility, respectively, in the same cellular conditions examined *in vitro* and *in vivo* (“inaccessible,” Figure 1C; white box). In summary, we show that a defined set of *Cdx2* CREs are transiently accessible at the time *Cdx2* is expressed in an *in vitro* model of SC development.

### Separate CREs control the onset versus maintenance of *Cdx2* expression

Having established that a distinct set of *Cdx2* CREs are transiently accessible, we set out to test directly the function of each CRE on the regulation of *Cdx2* during posterior body formation using a previously established ESC *in vitro* system to model SC or paraxial mesoderm development.^24^^,^^57^ We engineered a suite of ESC lines that lacked individual CREs corresponding to either *transient*, *constitutive*, or *inaccessible* regions using CRISPR-Cas9-mediated genome editing (Figures 1D and S1C). Wild-type (WT) versus CRISPR mutant ESC lines were then differentiated toward CEpiL cells that express *Cdx2* in response to a brief pulse of basic fibroblast growth factor (bFGF) and the glycogen synthase kinase-3 inhibitor, CHIR99021 (CHIR).^24^^,^^31^^,^^55^^,^^60^ Anterior epiblast-like (AEpiL) cells, which do not express *Cdx2*, were used as a control and induced by exposure to bFGF alone^31^^,^^32^^,^^55^^,^^57^ (Figures 1D and S1C). *Cdx2* induction was assayed by RT-qPCR and immunofluorescence (IF), together with flow cytometry, to investigate CDX2 in a quantitative and single-cell manner. The expression of CDX2 remained indistinguishable between WT cells and cells lacking either the *5′*, the *Iee* (Figures 1E–1G), or the *Tee* (Figures S1D–S1F) CRE (referred to as *Δ5′*, *ΔIee*, and *ΔTee* cells, respectively). By contrast, removal of the *Intron1* CRE severely impaired the induction of *Cdx2*, as demonstrated at the transcript (Figure 1E) and protein level (Figures 1F and 1G). Impaired induction of CDX2 was also recapitulated by the removal of the promoter for *Cdx2* (Figures S1D–S1F). This demonstrates that the intronic CRE plays an indispensable role in the induction of *Cdx2* in CEpiL cells. These data demonstrate that the removal of the *intron1* CRE is sufficient to block *Cdx2* induction in CEpiL cells, despite the presence of several alternative and accessible regulatory regions at the *Cdx2* locus, such as the *Iee* or the *5′* CRE (Figure 1C).

**Figure 2:**
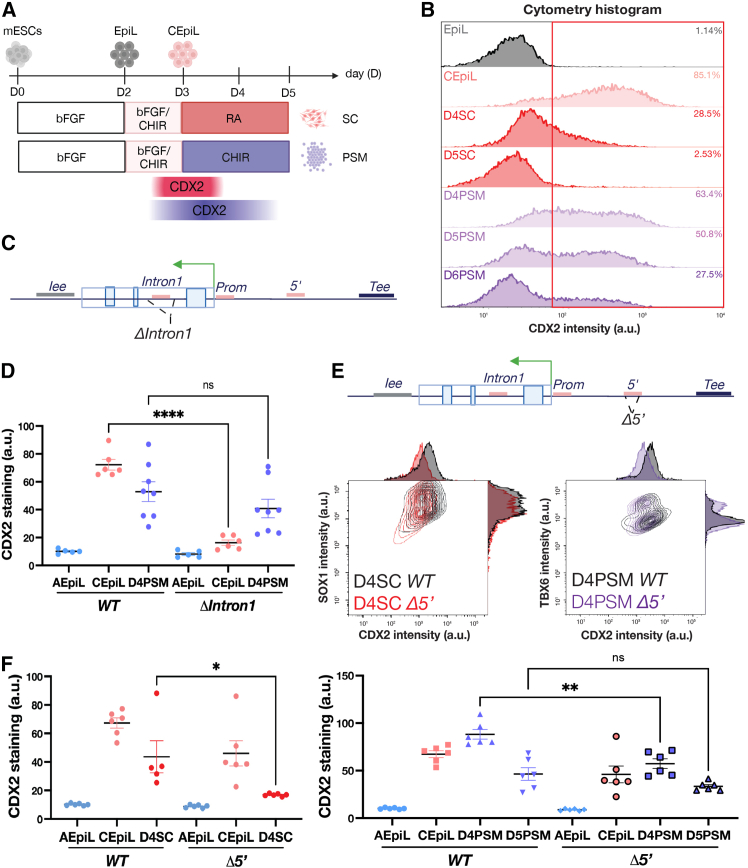
*Cdx2* is transiently maintained in CEpi derivatives via an alternative *5′* CRE (A) Schematic of the SC or PSM progenitor differentiation highlighting the transient expression of *Cdx2*. (B) Representative flow cytometry histogram for CDX2 in WT cells showing CDX2 detected in PSM conditions until day 5, versus day 4 in SC. (C) Schematic of the CRISPR-targeted element lacking in *ΔIntron1* ESCs. (D) Flow cytometry for CDX2 demonstrates WT and *ΔIntron1* cells display no differences by day 4 in PSM conditions. (E) Schematic of the CRISPR-targeted element lacking in *Δ5'* ESCs and representative flow cytometry contour and histogram plot for CDX2 and SOX1 or TBX6 in indicated conditions from *WT* (black) and *Δ5*′ (red and purple) cells. (F) CDX2 levels assessed by flow cytometry show a significant decrease of CDX2 in day 4 SC (red) and in day 4 PSM (purple) *Δ5*′ cells. (A), (C), and (E) created with BioRender.com. Data are represented as mean ± SEM. AEpiL, anterior epiblast-like; CEpiL, caudal epiblast-like; EpiL, epiblast-like; PSM, presomitic mesoderm; SC, spinal cord. ∗*p* value < 0.05, ∗∗*p* value < 0.01, ∗∗∗∗*p* value < 0.0001.

Having established that *Intron1* is indispensable for *Cdx2* induction in CEpiL cells, we tested whether *Intron1* was required for *Cdx2* expression in alternative cell types. To assess this, we differentiated ESCs under paraxial PSM conditions (Figure 2A), in which CDX2 is sustained for a longer time period (days 3–5) relative to SC progenitors (days 3–4) (Figures 2A and 2B). Despite an initial loss of *Cdx2* in CEpiL cells lacking *Intron1*, the expression of CDX2 is recovered at day 4 to levels comparable to WT cells in PSM conditions (Figures 2C and 2D; day 4 PSM condition). These data demonstrate that *Intron1* plays a cell-type-specific role in regulating *Cdx2* and suggest that an alternative CRE may be responsible for *Cdx2* expression in PSM conditions.

The *5′* CRE demonstrates transient accessibility and is also preferentially bound by CDX2 in CEpiL cells (Figure S2D). Under CEpiL conditions, ESCs lacking the *5′* CRE (Figure 2E) expressed CDX2 at comparable levels to WT cells (Figure 2F). However, *Δ5′* cells displayed a reduction in CDX2 expression at day 4 in both SC and PSM conditions, relative to WT cells (Figures 2E and 2F). RT-qPCR primers designed to detect spliced versus nascent *Cdx2* transcripts confirmed that a reduction in *Cdx2* was detectable at the level of transcription (Figure S2C). This suggests that *Cdx2* expression in derivatives of the CEpiL cells is dependent on the *5′* CRE, and, upon its removal, *Cdx2* is rapidly downregulated, decreasing the duration of expression (Figure S2C). Taken together, transient chromatin accessibility changes can be used to predict key regulatory elements that play nonredundant roles in the onset of *Cdx2* expression in CEpiL cells (*Intron1*) versus SC or paraxial mesoderm (*5′*) progenitors.

### The duration of *Cdx2* is regulated by separate subregions within the intronic CRE

**Figure 3:**
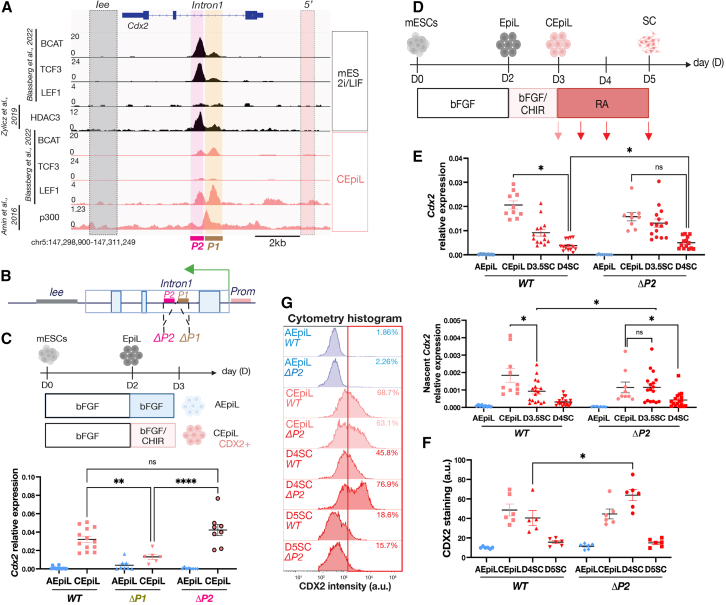
Cells lacking *P2* transiently prolong *Cdx2* in SC progenitors (A) ChIP-seq signal in naive ESCs (black) versus CEpiL cells (pink) from indicated studies, highlighting context-specific binding at *P1* and *P2*, adjacent regions located within the *Intron1* element but not the *Iee* or *5′* element. *P1* is occupied by LEF1 and p300, while CTNNB1 (βCAT), TCF3, and HDAC3 occupy *P2*. (B) Schematic illustrating the deleted region in *ΔP1* or *ΔP2* cells. (C) Relative expression (RT-qPCR) for *Cdx2* in indicated conditions from *ΔP1*, *ΔP2*, and *WT* cells demonstrates that *Cdx2* is induced in the absence of *P2* but not *P1*. (D) Schematic of SC differentiation highlighting the time points assayed (red arrows). (E) RT-qPCR for spliced and nascent *Cdx2* levels demonstrates that *ΔP2* cells fail to downregulate nascent transcription at day 3.5. (F and G) CDX2 levels (F) assessed by flow cytometry and representative cytometry histogram (G) for CDX2 show a population of CDX2-positive cells in *ΔP2*, unlike *WT* SC progenitors. (B), (C), and (D) created with BioRender.com. Data are represented as mean ± SEM. AEpiL, anterior epiblast-like; CEpiL, caudal epiblast-like; EpiL, epiblast-like; SC, spinal cord. ∗*p* value < 0.05, ∗∗*p* value < 0.01, ∗∗∗*p* value < 0.001.

To investigate what determines CRE usage in different cellular conditions, we sought to define what factors are responsible for CRE activity. CEpiL cells require active WNT signaling conditions to express *Cdx2*^9^^,^^31^^,^^32^^,^^55^ (Figure 1D). We therefore examined the chromatin immunoprecipitation sequencing (ChIP-seq) signal of several WNT effectors in naive mouse pluripotent ESCs versus CEpiL cells (Figure 3A), distinct cellular conditions that respectively repress or promote *Cdx2* expression.^57^ Analysis of these data revealed that despite the presence of TCF/LEF sites in multiple, accessible CREs (Figure 1C), the occupancy of WNT effectors is highly selective. CTNNB1 (βCAT), LEF1, and TCF3 are exclusively occupying the intronic CRE in both conditions (Figure 3A). Further examination of the intronic CRE revealed that WNT effectors occupy two distinct subregions within the *Intron1* CRE (labeled *P1* and *P2*; Figure 3A), depending on the cellular conditions. In naive pluripotency conditions, in which *Cdx2* is repressed, *P2* is occupied by CTNNB1 and TCF3. By contrast, the expression of *Cdx2* in CEpiL cells coincides with LEF1 preferentially occupying *P1*, while TCF3 and, to a lesser extent, CTNNB1 are depleted at *P2*.

We hypothesized from these data that *P1* and *P2* may mediate opposing regulatory functions that favor activation (at *P1*) versus repression (at *P2*) of *Cdx2*. Consistent with this hypothesis, the histone deacetylase 3 (HDAC3) preferentially accumulates at *P2* in naive pluripotency conditions,^61^ in contrast to the transcriptional co-activator p300, which occupies *P1* in CEpiL cells.^9^ These defined subregions contrast with the relatively broad deposition of H3K27me3 detected at *Cdx2* in both anterior and caudal (*Cdx2* expressing) epiblast tissues *in vivo*^62^ (Figure S3A), in addition to both ESCs and SC progenitors engineered *in vitro*^33^^,^^63^ (Figure S3B). To test the possibility that *P1* and *P2* perform separate regulatory functions, we generated ESCs lacking either *P1* or *P2* (Figure 3B) and directed their differentiation into CEpiL cells (Figure 3C). ESCs lacking *P1*, a region encompassing ∼225 bp, recapitulated the effect of removing the entire intronic CRE (990 bp): *Cdx2* induction was impaired despite exposure to active WNT signaling conditions (Figure 3C). By contrast, in the absence of *P2*, cells maintain the ability to induce *Cdx2* (Figure 3C). Strikingly, in SC conditions (Figure 3D), *P2*-lacking ESCs prolong *Cdx2* expression, with higher levels of spliced and nascent transcript (Figure 3E) detected transiently at day 3.5. By contrast, nascent transcription was comparable in the presence or absence of *P2* at day 4, while *P2*-lacking ESCs showed an increase in spliced transcript (Figure 3E) and higher levels of protein relative to control cells (Figures 3F and 3G). In summary, the data indicate that defined subregions within a single intron mediate opposing regulatory outcomes on *Cdx2* and identify *P2* as a critical region involved in the extinction of *Cdx2* in SC progenitors.

### RA nuclear receptor composition dictates regulatory element activity

**Figure 4:**
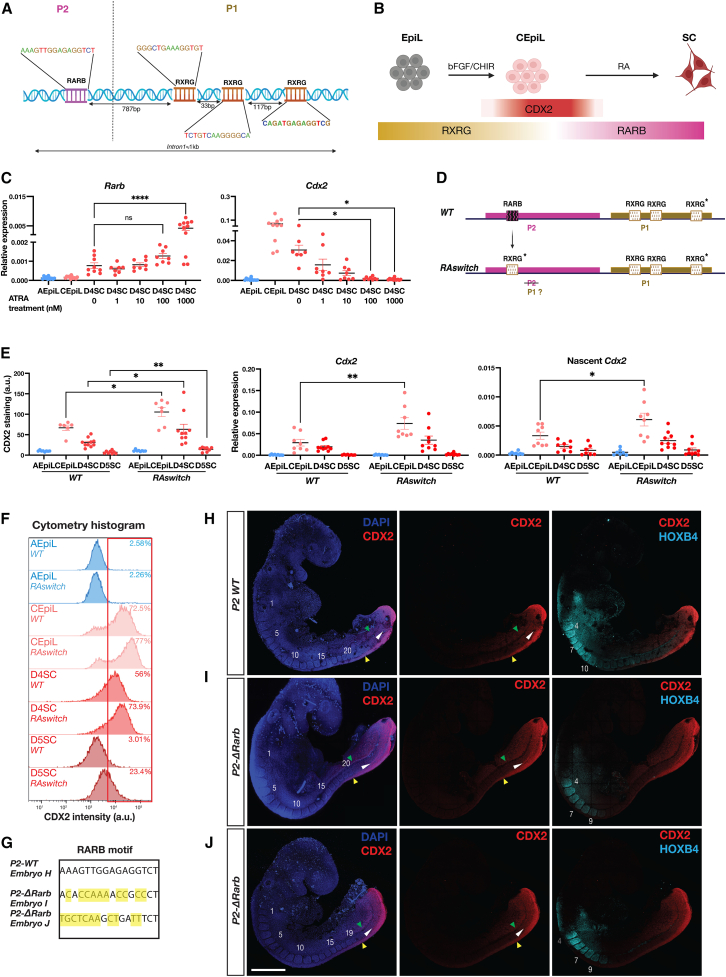
RARE motif switching controls *Cdx2* attenuator activity (A and B) Schematic of the RARE binding motifs distributed within *P1* and *P2* at *Intron1* and summary of the RA nuclear receptor expression changes observed as mESCs differentiate into SC progenitors.^24^^,^^31^ (C) Relative expression (RT-qPCR) for *Rarb* and *Cdx2* in cells exposed to increasing amounts of ATRA shows an increase in *Rarb* at 1 μM coinciding with a significant reduction in *Cdx2* in day 4 SC cells versus no ATRA treatment control. (D) *RAswitch* ESCs harbor a single copy of RXRG (asterisk) instead of an RARB motif at *P2*. (E) Quantification of CDX2 levels assessed by flow cytometry shows a significant increase in expression levels in *RAswitch* cells compared with *WT*, while relative expression (RT-qPCR) for *Cdx2* or nascent *Cdx2* shows a significant increase only in *RAswitch* CEpiL cells compared with *WT*. (F) Representative flow cytometry histogram for CDX2, related to (E). (G) Resulting genotypes in embryos presented in (H)–(J) following CRISPR-Cas9 in fertilized zygotes highlighting mismatches to the WT RARB motif. (H–J) Whole-mount IF shows broader detection of CDX2 (red) in the tailbud of *P2-ΔRarb* mutants (I and J) versus the control (H) at somite-matched stages. WT *Rarb* embryos display a caudal limit of HOXB4 that extends to somite 10 (H), versus a caudal limit at somite 9 in *P2-ΔRarb* mutants (I and J). Abnormal tailbud morphology is most apparent in the *P2-ΔRarb* mutant presented in (J). Somites are numbered in white; green, white, and yellow arrows indicate the approximate anterior limit of CDX2 expression in the lateral plate mesoderm, PSM, and neural tube, respectively. Scale bar represents 500 μm. (A), (B), and (D) were created with BioRender.com. Data are represented as mean ± SEM. ∗*p* value < 0.05, ∗∗*p* value < 0.01, ∗∗∗∗*p* value < 0.0001.

Having identified two adjacent regions harboring similar TCF/LEF motifs (Table S3), with opposing regulatory outcomes on *Cdx2*, we next asked what factors recruited to *P1* and *P2* could explain their functional differences. We performed motif analysis to predict TFs occupying *P1* and *P2* (Table S3). From this analysis, we recovered SOX and TCF/LEF sites at both *P1* and *P2*, consistent with their known occupancy at these sites^57^ (Figures 3A and S4E). In addition, we detected a striking difference in the composition of RAR versus RXR RA response elements (RAREs), recognized by the RA family of nuclear receptor TFs (Figures 4A, S4A, and S4B). Within *P1*, three separate, conserved RXRG motifs were detected (Figures 4A and S4A). By contrast, *P2* lacked any recognizable RXRG motifs and instead contained a single, rodent-specific RARB motif (Figures 4A, S4A, and S4B). In contrast to *Rarg*, which is detected throughout the differentiation, *Rxrg* levels drop while *Rarb* levels rise in single cells as they progress from a CEpiL to SC identity^24^ (Figure 4B). This raises the possibility that the combination of nuclear receptor subtypes present in different cell types^24^^,^^35^^,^^64^^,^^65^^,^^66^ plays a central role in regulating *Cdx2* expression.

Consistent with this view, CEpiL cells treated with increasing amounts of all-*trans*-RA (ATRA) display increased levels of *Rarb* in the resulting SC progenitors, while *Cdx2* was reduced (Figure 4C). Having established that increased levels of *Rarb* correspond to lower levels of *Cdx2* in SC progenitors, we sought to verify whether the repressive activity of *P2* relies on the presence of the RARB motif in the region. We generated ESCs in which 75% of the RARB motif present in *P2* was disrupted (Figure S4C highlighted in yellow). Mimicking the *ΔP2* cells (Figure 3E), the resulting *ΔRarb* day 4 SC cells showed an increase in *Cdx2* transcript and protein (Figure S4D) levels relative to *WT* day 4 SC cells.

These findings raise the possibility that variation in RARE motifs dictates regulatory element function. To test the hypothesis that motif composition underpins the functional differences between *P1* and *P2*, we generated ESCs in which the single RARB motif present in *P2* was replaced with a single copy of the RXRG motif, identical in sequence to the highest-affinity RXRG site detected in *P1* (Figure 4D; RXRG site labeled with an asterisk). The resulting *RAswitch* ESCs, which harbor an additional RXRG site compared with control cells, displayed an increase in *Cdx2* levels in CEpiL cells, detected at both the transcript and protein level (Figures 4E and 4F, in light pink). In addition, as *RAswitch* cells progressed to an SC identity, CDX2 expression was maintained, in contrast to control cells, which instead began downregulating *Cdx2*, demonstrating the vital role of the RARE subtype at *P2* to ensure the appropriate offset of *Cdx2* in SC progenitors (Figures 4E and 4F, in red). Taken together, the data demonstrate that the total number and composition of RAREs within *P2* determine its regulatory effect on *Cdx2*.

As the onset and termination of *Cdx2* is mediated through the intronic CRE, also bound by several WNT effectors, how do alternative RA nuclear receptors facilitate *Cdx2* transcriptional control (Figure 3A)? Tight control over the level of SOX2 in CEpiL cells is required for *Cdx2* expression and correlates with genome-wide redistribution of several TCF/LEF effectors, both at the *Cdx2* locus and at a genome-wide scale^57^ (Figure S4E). To probe potential interactions between RARb or RXRg and LEF1 or SOX2, we used AlphaFold-Multimer^67^ to perform *in silico* predictions of possible protein-protein complexes (Figure S4F). These simulations provide evidence that both RARB and RXRG can interact with LEF1 and SOX2. In line with this, ChIP-qPCR experiments showed that the enrichment of RARB at *P2* is reduced, together with LEF1 and SOX2 (Figures S4H and S4I), in day 4 SC *RAswitch* cells that lack the RARB motif at *P2*. By contrast, similar levels of LEF1 and SOX2 are detected across control and *RAswitch* cells (Figure S4I) at *P1*. Taken together, these findings support the hypothesis that, as cells adopt an SC identity, the loss of *Cdx2* coincides with the accumulation of LEF1 and SOX2 at *P2* in an RARB-dependent manner.

### P2 is indispensable for posterior body formation

Having established a model of *Cdx2* regulatory control during posterior body formation *in vitro*, we sought to validate these findings *in vivo*. To this end, we attempted to perturb *Cdx2* by disrupting the RARB site within *P2* in mouse embryos. We reasoned that if *P2* limits the duration of *Cdx2* during posterior body formation (Figures 3E–3G), disruptions to the RARB site alone would be expected to transiently prolong *Cdx2* expression, an effect known to perturb tailbud morphology and *Hox* gene expression boundaries in mouse embryos.^2^^,^^11^ We used CRISPR-Cas9, together with the same gRNA pairs used *in vitro*, to remove *P2* (Δ*P2*) or to disrupt directly the RARB site in fertilized zygotes and collected resulting somite-matched transient transgenic embryos at mid-gestation (Table S2). *P2-ΔRarb* CRISPR mutants recovered at ∼E9 harbored mutations and deletions within the *P2* region (Figure 4G). The RARB site was disrupted in mutants (Figures 4G, 4I, and 4J) relative to control littermates that harbored an entirely intact *P2* CRE (*WT P2*; Figures 4G and 4H).

Whole embryo IF and imaging confirmed that P2-*WT* embryos recapitulated the known endogenous expression pattern of CDX2,^3^^,^^9^ which is restricted to the tailbud and the caudal-most aspect of the neural tube at this stage (Figure 4H). Strikingly, the *P2-ΔRarb* CRISPR mutant embryos displayed a rostral expansion in CDX2, most notably in the mesoderm (Figures 4I and 4J; green and white arrows). In addition, regional identity was disrupted in the somites. Mutant embryos displayed a caudal limit of HOXB4 that extended to somite 9, in contrast to the control, which reached a more caudal position, up to somite 10 (Figure 4; compare Figures 4H–4J). Mutant embryos also displayed abnormal tailbud morphology, reminiscent of the phenotype resulting from prolonged *Cdx2* expression in mouse embryos.^2^ These data provide evidence that *P2* is required to restrict the activity of CDX2 *in vivo* and pinpoint the RARB motif as a critical site that operates during posterior body formation.

## Discussion

### Multiple, nonredundant CREs coordinate *Cdx2* expression during development

Using an *in vitro* model of posterior body development, we demonstrate that multiple, functionally discrete CREs convert extrinsic cues into a finite window of expression. These data support the idea proposed by previous enhancer reporter experiments that multiple CREs located proximally to the promoter regulate *Cdx2*.^53^ Furthermore, our data extend these findings by demonstrating the functional specificity of CREs during development: *Intron1* is required for induction in CEpiL conditions (Figures 1E–1G), in contrast to the *5′* CRE, which is not required in this context but is indispensable at later stages to maintain *Cdx2* transiently in SC or paraxial mesoderm progenitors (Figure 2F). Furthermore, the occupancy of CDX2 at the *5′* CRE suggests that this element may perform its maintenance function at least in part via a positive feedback loop (Figure S2D). Previous studies also proposed that a silencer may regulate the caudal expression pattern of *Cdx2*.^53^ Here, we resolve a minimal region residing within *Intron1* (*P2*; Figure 3A) required to limit the duration of *Cdx2* in SC progenitors (Figures 3E–3G), and validate its requirements for appropriate CDX2 activity *in vivo* (Figures 4H–4J). By contrast, previous studies demonstrate that a fragment containing *P2* displays enhancer activity in transgenic mouse reporter assays.^53^^,^^59^ Although we cannot exclude the possibility that *P2* displays enhancer activity in an alternative context and thus may act as a bifunctional element,^68^^,^^69^ our findings highlight that targeted base pair substitutions at CREs in their native context can aid in the identification of regulatory regions that include repressive elements.

Our results confirm that individual CREs perform indispensable roles since single CRE deletions are sufficient to perturb the expression window and cannot be compensated for by the presence of alternative and accessible CREs (Figures 1E–1G, 2E, 2F, and 3C–3G). The requirement for several, functionally distinct CREs may ensure robustness in gene expression.^70^^,^^71^^,^^72^ Consistent with this view, removal of the *5′* or *P1* region has a clear but limited effect on *Cdx2* expression, potentially due to the presence of additional CREs that are yet to be resolved. In addition, *P2* extends the expression window in SC progenitors, yet *Cdx2* is eventually extinguished in these cells (Figures 3E–3G). The presence of additional, potentially long-range CREs likely explains this effect. Evidence of long-range CREs impacting *Cdx2* expression has been previously described in an alternative cellular context, in B cell acute lymphoblastic leukemia patients.^51^^,^^52^ Such long-range CREs may also buffer fluctuations of extrinsic signaling, as observed in the zebrafish neural plate border for *Zic3* expression^73^ and more recently in mouse embryos.^70^^,^^74^

### Motif composition dictates attenuator function

In this study, *P2* represses *Cdx2* in a context-specific manner, as its removal transiently alters the level of nascent transcription in SC progenitors but not CEpi conditions (Figure 3E). Whether *P2* mediates any part of its repressive function at the level of splicing or transcript stability remains to be determined. As *P2* represses *Cdx2* in a limited manner, we refer to this element as an “attenuator.” In contrast to enhancers, relatively few repressive CREs have been identified and functionally validated during development, especially in mammals.^75^ As functional validation of repressive elements is challenging to perform at scale,^76^ the mechanisms that distinguish repressive versus activating elements remain to be elucidated.

*Cdx2* is expressed in response to active WNT signaling conditions, and ChIP-seq against LEF1 demonstrates its preferential accumulation at *P1* (Figure 3A). However, TCF/LEF binding motifs are also present in multiple *Cdx2* CREs, yet, despite their accessibility, these sites do not compensate for the intronic CRE upon its removal in *ΔIntron1* or *ΔP1* cells. These findings suggest that chromatin accessibility is not sufficient to predict enhancer function at CREs,^58^ which indicates that an additional mechanism is involved. Recent findings indicate that the specificity of TCF/LEF binding in the genome is driven by context-specific TFs^77^ and their level of expression.^57^ In CEpiL cells, SOX2 levels dictate the genome-wide occupancy of several WNT effectors, including the occupancy of LEF1 and CTNNB1 at the *Cdx2* intronic CRE.^57^ Moreover, the activity of the intronic CRE requires SOX2 binding sites,^57^ consistent with the view that the recruitment of WNT effectors is driven by cooperation between cell identity-specific TFs. As *P1* and *P2* both harbor SOX2 binding sites and can be occupied by SOX2,^57^ an additional molecular determinant must govern *P1* versus *P2* function.

In this study, we provide evidence that regulatory element function is dependent on motif composition. We demonstrate that *P2* can be converted into an enhancer through a single motif switch from RARB to RXRG (Figures 4E and 4F). This switch disrupts RARB enrichment and the recruitment of SOX2/LEF1 at *P2*. As RARB and RXRG can each interact with SOX2 and LEF1 *in silico*, these findings suggest that the regulatory function of *P2* is driven by RA nuclear receptor subtypes (Figure S4F). Dissection of individual elements in different species demonstrates that motif composition can dictate silencer function in *Drosophila*.^78^^,^^79^ In mammalian genomes, the same CRE can operate as an enhancer or silencer, a function that changes depending on the cellular context.^80^^,^^81^^,^^82^^,^^83^^,^^84^ Our results demonstrate that a single nuclear receptor motif switch is sufficient to change the function of a given CRE without altering the cellular conditions. These findings support the view that TF engagement contributes to the functional versatility of CREs across different cell types.^58^^,^^81^^,^^84^

### Repressive mechanisms operating during posterior body development

Although RA is a known major determinant of posterior body formation,^3^^,^^24^^,^^27^^,^^35^^,^^36^^,^^85^^,^^86^^,^^87^ its mechanism of action is not fully understood. Among the predicted 14,000 potential RAREs in the mouse genome,^88^ only a handful have been experimentally validated, including both enhancers^89^^,^^90^ and silencers.^91^^,^^92^ However, what underpins enhancer or silencer activity at individual RAREs remains unclear.^90^ Here, we identify activating and repressive RAREs for *Cdx2* that suggest RARB and RXRG may exert opposing regulatory roles. Furthermore, we validate that the RARB motif we have identified is occupied by the nuclear receptor RARB and demonstrate that variation in RARE motifs impacts RARB occupancy and regulatory element function. The data suggest that differences in nuclear receptor recruitment at individual RAREs may underpin the pleiotropic role of RA during development.^93^

Recruitment of cofactors is likely to impart distinct functions at RAREs. Consistent with this view, the occupancy of distinct RA nuclear receptors is associated with changes in cofactor recruitment. RARG recruits the transcriptional co-activators pCIP/p300 to an RARE regulating *Hoxa1*, whereas the occupancy of RARB/RARA abolishes p300 and instead promotes the recruitment of the Polycomb subunit SUZ12.^89^ In addition, NCOR1/2,^94^ HDAC,^92^^,^^95^ and Polycomb repressive complexes (PRCs)^89^^,^^92^ are recruited in the vicinity of RAREs associated with transcriptional repression in response to RA signaling. PRC recruitment can also impact chromatin compaction,^96^^,^^97^ yet how these events relate to nuclear receptor engagement at defined CREs remains unclear. Our findings indicate that HDAC3 accumulates at *P2* in repressive conditions (Figure 3A); how this relates to RARB is unresolved. By contrast, PRC is deposited in a widespread manner at *Cdx2* (Figure S3), as commonly observed at developmental genes.^33^^,^^98^^,^^99^ In summary, multiple mechanisms are likely to explain how nuclear receptor subtypes exert regulatory roles during development.

Since previously predicted^75^^,^^100^ and experimentally validated^101^^,^^102^^,^^103^ silencers are located relatively close to the transcription start site of genes and can be found adjacent to enhancers,^100^^,^^101^ short-range gene silencing mechanisms may represent a more general principle of gene regulation during development. Consistent with this view, *Cdx1* is regulated by a silencer located ∼400 bp upstream of the promoter and ∼500 bp away from an enhancer.^101^ Physical obstruction of individual enhancers or their interaction with the promoter could silence gene expression.^39^

CDX factors display a graded expression profile along the rostrocaudal axis,^19^ which, in turn, plays a central role in constraining regional identity through the regulation of *Hox* genes.^2^ That CDX factors contain conserved regulatory elements and play a caudalizing role in several species^8^^,^^104^^,^^105^^,^^106^^,^^107^^,^^108^ suggests that the regulatory principles governing their transient expression may underpin body plan organization across multiple bilaterian animals.

### Limitations of the study

In the current work, how *P2* attenuates *Cdx2* is not resolved. Future studies will elucidate how the occupancy of RARB at *P2* impacts transcription, broadening our understanding of the gene regulatory mechanisms operating during mammalian development.

## Resource availability

### Lead contact

Further information and requests for resources and reagents should be directed to and will be fulfilled by the lead contact, Vicki Metzis.

### Materials availability

Murine ESC lines generated in this study are available upon request.

### Data and code availability

Accession numbers of the reanalyzed sequencing dataset are provided in the STAR Methods section. All original code has been deposited at https://github.com/da-bar/cdx2_transient_expression. Any additional information required to reanalyze the data reported in this paper is available from the lead contact upon request.

## Acknowledgments

We thank James Briscoe, Joaquina Delas, Matthias Merkenschlager, Teresa Rayon, Kate Storey, Juan M. Vaquerizas, and all lab members for comments on the manuscript. We are grateful to Tristan Rodriguez for advice on the generation of transgenic embryos. For support, training, and access to equipment, we thank James Elliot from the LMS/NIHR Imperial Biomedical Research Centre Flow Cytometry Facility, the staff at the Central Biomedical Services unit at Imperial College London, Zoe Webster at the LMS transgenic facility, and Dirk Dormann at the LMS microscopy facility. This work was supported by core funding to the Laboratory of Medical Sciences from the Medical Research Council, a Sir Henry Dale Fellowship awarded to V.M., jointly funded by the Wellcome Trust and the Royal Society (grant number 218536/Z/19/Z), and a Small-scale Researcher Mobility Scheme between the UK and Croatia (011-31/1-2024.pm) to D.B. This work was funded by the European Union – NextGenerationEU grant NPOO.C3.2.R2-I1.06.0060.

## Author contributions

I.A. and V.M. conceived the project, designed the experiments, interpreted the data, and wrote the manuscript. I.A. performed the experiments and data analysis, with assistance from S.Q.X. D.B. performed data analysis and together with B.L. interpreted the data. B.M. performed microinjections and embryo transfers. M.P. performed data analysis. All authors revised the manuscript.

## Declaration of interests

The authors declare no competing interests.

## STAR★Methods

### Key resources table

**Table:** 

REAGENT or RESOURCE	SOURCE	IDENTIFIER
**Antibodies**		

Mouse monoclonal anti-CDX2 – clone 88	Abcam	Cat#ab157524; RRID: AB_2721036
Mouse monoclonal anti-CDX2 – clone 88	Gentaur	Cat#MU392A-5UC; RRID: AB_2923402
Rabbit monoclonal anti-CDX2	Abcam	Cat#ab76541; RRID: AB_1523334
Goat polyclonal anti-SOX1	R&D	Cat#AF3369; RRID: AB_2239879
Rat monoclonal I12 anti-HOXB4	DHSB	Cat#AB_2119288; RRID: AB_2119288
Goat polyclonal anti-BRACHYURY	R&D	Cat#AF2085; RRID: AB_2200235
Goat polyclonal anti-SOX2	R&D	Cat#AF2018; RRID: AB_355110
Goat polyclonal anti-TBX6	R&D	Cat#AF4744; RRID: AB_2200834
Rabbit polyclonal anti-RARB	Invitrogen	Cat#PA1-811; RRID: AB_2253602
Mouse monoclonal anti-LEF1	Millipore	Cat#17-604; RRID: AB_916350
anti-mouse AlexaFluor 488	Thermo Fisher	Cat#A21202; RRID: AB_101607
anti-rabbit AlexaFluor 488	Thermo Fisher	Cat#A21206; RRID: AB_2535792
anti-goat AlexaFluor 488	Thermo Fisher	Cat#A11055; RRID: AB_2534102
anti-rabbit AlexaFluor 568	Thermo Fisher	Cat#A10042; RRID: AB_2534017
anti-mouse AlexaFluor 568	Thermo Fisher	Cat#A10037; RRID: AB_11180865
anti-goat AlexaFluor 647	Thermo Fisher	Cat#A21447; RRID: AB_2535864
Anti-rat AlexaFluor 488	Thermo Fisher	Cat#A21208; RRID: AB_2535794
Anti-rat AlexaFluor 647	Thermo Fisher	Cat#A78947; RRID: AB_2910635

**Bacterial and virus strains**		

Electro-competent DH5alpha cells	Thermo Scientific	Cat#EC0112

**Chemicals, peptides, and recombinant proteins**		

Dulbecco’s Modified Eagle Medium (DMEM) - Knock OUT	Gibco	Cat#10829-018
ESGRO Mouse LIF Medium	Merck Millipore	Cat#ESG1107
DMEM F12	Gibco	Cat#21331-020
Neurobasal medium	Gibco	Cat#21103-049
L-Glutamine	Gibco	Cat#25030-024
Trypsin-EDTA 0.05%	Gibco	Cat#25300-054
StemPro Accutase	Gibco	Cat#A11105-01
BSA	Sigma	Cat#A7979
N2 Supplement	Gibco	Cat#17502-001
B27 Supplement	Gibco	Cat#A35828-01
B27 Supplement, minus vitamin A	Gibco	Cat#A3353501
Gelatin	Sigma	Cat#G1393-20ML
b-mercaptoethanol	Gibco	Cat#21985-023
Recombinant bFGF	PeproTech	Cat#100-18B-10uG
CHIR99021	Axon	Cat#1386
All-*trans*-Retinoic Acid (ATRA or RA)	Sigma	Cat#R2625-50MG
Live/dead fixable blue dead cells stain kit for UV excitation	Thermo Fisher	Cat#L34961A
RNAse-free DNase I	Qiagen	Cat#79254
Superscript III reverse transcriptase	Thermo Fisher	Cat#18080-051
PowerUp SYBR-Green Mastermix	Thermo Fisher	Cat#A25742
Puromycin	Gibco	Cat#A11138-03
ES Fetal Bovine Serum (FBS)	Pan Biotech	Cat#P30-2602; Lot P200304ES
Penicillin/Streptomycin	Gibco	Cat#15140122
Non-essential amino acids	Gibco	Cat#11140-050
Phosphate Buffer Saline (PBS)	Pan Biotech	Cat#P0436500
Paraformaldehyde (PFA)	ThermoScientific	Cat#28908
Triton-X100	Sigma	Cat#T8787-250ML
ProLong Gold antifade reagent	Invitrogen	Cat#P36930
GlutaMAX	Gibco	Cat#35050-061
Di(N-succinimidyl) glutarate (DSG)	Sigma	Cat#80424-5MG-F
Glycine	Sigma	Cat#G7126-1KG
PBS with CaCl_2_/MgCl_2_	Sigma	Cat#D8662
DMSO	Sigma	Cat#D2650-100ML
Dynabeads Protein G	Thermo Fisher	Cat#10004D
SDS	Thermo Fisher	Cat#BP1311-200
EDTA	Corning	Cat#46-034-CI
EGTA	Thermo Fisher	Cat#J60767.AD
Hepes	Sigma	Cat#H0887-100ML
NaCl 5M solution	Lonza	Cat#51202
Protease Inhibitor cocktail (PI)	Sigma	Cat#P8340-5ML
Sodium Bicarbonate (NaHCO3)	Sigma	Cat#S6297-250G
Sodium Deoxycholate	Sigma	Cat#30970-25G
NP-40	Sigma	Cat#I8896-100ML
LiCl	Sigma	Cat#L4408-100G
PureLink RNase A (20mg/mL)	Invitrogen	Cat #12091021
Proteinase K, ChIP-grade	Thermo Fisher	Cat#26160

**Critical commercial assays**		

QIAGEN RNeasy	Qiagen	Cat#74106
PureLink genomic DNA extraction Kit	Invitrogen	Cat#K182002
Mouse Embryonic Stem Cell nucleofector kit	Lonza	Cat#VPH-1001
Qiaquick PCR Purification Kit	Qiagen	Cat#28106

**Experimental models: Cell lines**		

Mus musculus (Male): HM1 *WT*	Doetschman et al.^109^	N/A
Mus musculus (Male): HM1 *ΔIee*	This study	N/A
Mus musculus (Male): HM1 *Δ5’*	This study	N/A
Mus musculus (Male): HM1 *ΔTee*	This study	N/A
Mus musculus (Male): HM1 *ΔP1*	This study	N/A
Mus musculus (Male): HM1 *ΔP2*	This study	N/A
Mus musculus (Male): HM1 *ΔIntron1*	This study	N/A
Mus musculus (Male): HM1 *ΔProm*	This study	N/A
Mus musculus (Male): HM1 *ΔRarb*	This study	N/A
Mus musculus (Male): HM1 *RAswitch*	This study	N/A

**Oligonucleotides**		

qPCR primers used in this study, see Table S1	This study	N/A
sgRNA sequence, genotyping primers and sequencing primers used to generate cell lines see Table S2	This study	N/A
sgRNA sequence used for in vivo targeting of the P2 CRE, see Table S2	This study	N/A

**Recombinant DNA**		

PX459; SpCas9-2A-Puro and single guide RNA	Ran et al.^110^	Cat#62988 (Addgene)

**Software and algorithms**		

nf-core chipseq pipeline	https://nf-co.re/chipseq Ewels et al.^111^	https://nf-co.re/chipseqhttps://doi.org/10.5281/zenodo.3240506
nf-core rnaseq pipeline	https://nf-co.re/rnaseq Ewels et al.^111^	https://doi.org/10.5281/zenodo.1400710
Fiji	Schindelin et al.^112^	https://imagej.net/software/fiji/
TFBStools version 1.40.0	Tan and Lenhard^113^	http://bioconductor.org/packages/TFBSTools/
IGV	Robinson et al.^114^	https://igv.org/

**Other**		

Donkey serum	Abcam	Cat#ab7475
35 mm high glass bottom imaging dish	Ibidi	Cat#81158
CellBind 6-well plate	Corning	Cat#3335
35 mm CellBind dish	Corning	Cat#3294
32 mm coverslips, no. 1.5 thickness	SLS	Cat#631-0162
100 mm CellBind dish	Corning	Cat#3296
Cell scrapper	VWR	Cat #734-2602
RNase-free tube	Ambion	Cat#AM12450
Diagenode tube for sonication	Diagenode	Cat#C30010010-300

### Experimental model and study participant details

#### Cells lines

All mouse ESC lines were cultured at 37 °C with 5% CO_2_, and were visually inspected on a daily basis. All mouse ESC lines were subject to mycoplasma testing on a monthly basis. All ESC lines used were derived from the XY HM1 line (129/Ola strain),^109^ which was used as the WT control. *ΔIntron1*, *Δ5’*, *ΔIee*, *ΔProm*, *ΔTee*, *ΔP1* and *ΔP2* lines were generated by electroporating pairs of CRISPR targeted to both extremities of the regions of interest (Table S2). After puromycin selection (at a concentration of 1.5 ug/mL), 10 clones were picked and expanded. gDNA was extracted using the PureLink kit (PureLink™ Genomic DNA) according to the manufacturer’s instructions. Clones were then genotyped (Table S2), validated for the deletion of the targeted region in both alleles by DNA sequencing. For most of the generated KO cell lines, we obtained similar results with a second clone (*ΔIntron1*, *ΔTee*, *ΔProm*, *ΔP2*, *ΔIee*).

The *RAswitch* and the *ΔRarb* lines were created using HDR recombinant oligos electroporated with the sgRNA guide (Table S2) and the Cas9 protein (#1081058) supplemented with the Alt-R Cas9 electroporation enhancer (#1075915) into HM1 cells. After recovery using the Alt-R HDR EnhancerV2 (#10007910), 10 clones were picked, expanded, and, genotyped (Table S2) and validated by DNA sequencing in a similar method as described above.

#### ESC culture and differentiation

All mouse ESCs were expanded on mitotically inactivated mouse embryonic fibroblasts (feeders) in ESC medium (DMEM knockout medium supplemented with 1.000U/ml LIF, 10% cell-culture-validated foetal bovine serum, and 2mM L-Glutamine). Data were obtained using low-passage cells (i.e. passaged no more than 10 times after thawing for a total number of 32 passages since derivation).

To differentiate mESCs into neural or paraxial presomitic mesoderm progenitors, ESCs were differentiated as previously described.^24^^,^^55^ Briefly, ESCs were dissociated with 0.05% trypsin, and plated on tissue-culture-treated plates for two sequential 20 minutes (mins) periods in ESC medium to separate them from their feeder layer cells, which adhere to the plastic. To start the differentiation, cells remaining in the supernatant were pelleted by centrifugation, counted, and resuspended in N2B27 medium containing 10 ng/ml bFGF, and 40 000 cells per 35 mm gelatin-coated CellBIND dish or 6-well plate (Corning) were plated. N2B27 medium contained a 1:1 ratio of DMEM/F12:Neurobasal medium (Gibco) supplemented with 0.5% N2 (Gibco), 1% B27 (Gibco), 2mM L-glutamine (Gibco), 40mg/ml BSA (Sigma), and 0.1mM 2-mercaptoethanol.

To generate Epiblast-like (EpiL) or Anterior Epiblast-like (AEpiL) cells, the cells were grown respectively for 2 and 3 days in N2B27 +10 ng/ml bFGF. To generate Caudal Epiblast-like (CEpiL) cells, cells were cultured with N2B27 + 10 ng/ml bFGF for 2 days, then N2B27 + 10 ng/ml bFGF +5 μM CHIR99021 for a further day. CEpiL cells were differentiated to spinal cord neural progenitors by continuing the differentiation up to day 5 in N2B27 media containing 10nM all-*trans*-retinoic acid (ATRA, 10nM). In the experiments presented in Figure 4C, CEpiL were either exposed to N2B27 alone for one day or exposed to concentrations of ATRA ranging from 1nM to 1μM.

To generate paraxial mesoderm progenitors, CEpiL cells (generated as described above) were exposed to 5μM CHIR (GSK3β inhibitor) for a further 2 days. For the paraxial mesoderm differentiation, a CEpiL were grown in N2B27 using B27 devoid of Vitamin A as previously described.^24^ Media was refreshed every day from day 2-5 for all experiments.

Details of key compounds are provided in the STAR Methods.

#### CRISPR mutant embryos

For generation of the *P2-ΔRarb* CRISPR mutants, gRNA sequences (Table S2) were ordered as oligonucleotides (Integrated DNA Technologies) together with recombinant Cas9 protein (Alt-R™ S.p. Cas9 Nuclease V3; 1081058). The sgRNA (at 25 ng/μl) and the Cas9 (at 75 ng/μl) were combined and microinjected into the pronuclei of one-cell embryos in two separate rounds of injection (Figure 4).

One-cell embryos obtained by super-ovulating 10 C57Bl/6 females with 50 IU of PMSg 48h hours before mating and with 50 IU of HCG on the day of mating were mated with C57Bl/6 stud males. 24h after mating embryos were harvested, cleaned and placed in culture media (KSOM) at 37°C. Each zygote was then microinjected into the pronuclei with the CRISPR/Cas9 complex. Microinjected zygotes were transferred back into recipient females (B6CBAF1; previously mated and plugged by vasectomised CD1 males) by embryo transfer procedure at 0.5dpc. All females were monitored daily in a Biological Support Unit. The recipient females were humanely killed and embryos were harvested at 9.5dpc.

After dissection and collection of amniotic tissue for genotyping, embryos were fixed in 4% paraformaldehyde in PBS for 90 mins at 4°C under gentle agitation, followed by two washes in PBS.

Embryos were genotyped using the HotSHOT DNA^115^ extraction protocol. Briefly, amnions were incubated at 95°C for 30 mins in 25uL alkaline lysis buffer before addition of 25uL neutralizing buffer and storage at 4°C. The *Intron1* fragment was amplified by PCR using the primers used for the genotyping of the *ΔIntron1* cell line (Table S2). After purification using the Qiagen PCR purification kit, the fragment was sequenced using the primer outlined in Table S2.

All animal procedures were performed by certified staff in the Imperial College London Central Biomedical Services Facility and all experiments were performed with ethical approval in accordance with the Animal (Scientific Procedures) Act 1986 with ethical approval under the UK Home Office project license PP2904879. Animals were housed in a 10-hour light, 14-hour dark cycle with access to food and water ad libitum in individually ventilated cages. The temperature was maintained at 21-24°C and 45-65% humidity. No distinction was made between male and female embryos during analysis.

### Method details

#### Immunofluorescence on cells

Cells were washed in PBS and fixed in 4% paraformaldehyde in PBS for 30 mins at 4°C, followed by three washes in PBS. Primary antibodies (STAR Methods) were applied overnight at 4°C diluted in filtered blocking solution (2% BSA diluted in PBST – 0.1% Triton X-100 diluted in PBS). Cells were washed for 5 mins three times in PBST and incubated with secondary antibodies (STAR Methods) at room temperature, for 90 mins. Cells were washed for 5 mins three times in PBST, incubated with DAPI for 15 mins in PBS and washed twice before mounting with a glass coverslip using Prolong Gold (Invitrogen) or kept in PBS for further imaging.

Cells were imaged on an inverted SP5 or upright SP5 II confocal microscope (Leica). Z stacks were acquired using the Leica LAS AF software and represented as maximum intensity projections using ImageJ software. The same settings were applied to all images. Images presented in Figures 1G and S1F are representative images of a minimum of three biological replicates.

#### Flow cytometry

Cells were washed in PBS and dissociated with Accutase (Gibco). Once detached, cells were collected, washed with PBS, and pelleted. Cells were resuspended in PBS supplemented with live dye (1/1000, Thermo Fisher) and kept in dark at 4° for 30 mins. Cells were pelleted, washed in PBS, pelleted, and resuspended in 4% paraformaldehyde in PBS. Following 15 mins incubation at 4°C, cells were centrifuged, resuspended in PBS, and stored at 4°C for future analysis.

On the day of flow cytometry, cells were transferred for staining in U-bottom 96-well plates. Samples were pelleted and resuspended in 50μl block media (2% BSA diluted in PBST). After 30 mins incubation at room temperature in the platform rocker, antibodies were added to the sample and incubated overnight at 4°C on a platform rocker. Details of primary and secondary antibodies are described in the STAR Methods. Cells were pelleted for 4 mins, washed in PBST, pelleted, and incubated in 50μl PBST supplemented with secondary antibodies (concentration: 1/500) in the dark for 2h at room temperature in the platform rocker. One additional wash was performed before acquisition on a SymphonyA3 (BD Biosciences) using FACSDiva. Analysis was performed using FlowJo.

#### RNA extraction, cDNA synthesis and RT-qPCR analysis

RNA used for real time quantitative PCR (RT-qPCR) was extracted from cells using a QIAGEN RNeasy kit in RLT buffer, following the manufacturer’s instructions. Extracts were digested with DNase I to eliminate genomic DNA.

First-strand cDNA synthesis was performed using Superscript III (Invitrogen) using random hexamers and was amplified using PowerUp SYBR-Green Mastermix (Applied Biosystems). RT-qPCR was performed using the Applied Biosystems QuantStudio Real Time PCR system and analysed with Applied Biosystems QuantStudio 12K Flex software. PCR primers were designed using the online PrimerBLAST design tool and validated (standard curve and melting curve) or taken from previously published papers. Primer sequences are detailed in Table S1. Two technical replicates were obtained for each sample and averaged before normalization and statistical analysis. Relative expression values for each gene were calculated by normalization against β-actin, using the delta–delta CT method. RT-qPCR analysis was performed on samples obtained from a minimum of three independent experiments for every primer pair analysed.

#### Embryo wholemount immunofluorescence

Embryos were permeabilized in 0.5% Triton X-100 diluted in PBS for 30 mins at room temperature under gentle agitation. After permeabilization, embryos were incubated in filtered block media (2% BSA and 4% donkey serum diluted in PBST) at room temperature for 2 hrs under gentle agitation. Primary antibodies (STAR Methods) were applied overnight at 4°C diluted in filtered block media under gentle agitation. The following morning, embryos were washed for 2 hrs in PBST, 4–5 times, at room temperature under gentle agitation and incubated in filtered block media overnight at 4°C under gentle agitation. Secondary antibodies (STAR Methods) were applied diluted in PBST (1/500) at room temperature for 90 mins in the dark under gentle agitation. After 10 mins PBST washes, embryos were incubated with DAPI (1/1000) in PBST in the dark under gentle agitation at room temperature for 30 mins.

After PBST washes, embryos were mounted in 1.5% low-melt agarose in p35 Ibidi plates before imaging using an inverted Leica DLS. Z stacks were acquired using the Leica LAS AF software and represented as maximum intensity projections using ImageJ software.

#### ChIP-qPCR

Adherent cells were washed three times with PBS, fixed with gentle agitation for 45 mins at room temperature with fresh 2mM di(N-succinimidyl) glutarate in PBS+ (DPBS with CaCl_2_/MgCl_2_), washed an additional three times with PBS+, then fixed for 10 mins at room temperature with 1% molecular-biology-grade paraformaldehyde in PBS+. Fixation was quenched by addition of 250 mM glycerine for 5 mins, followed by three additional washing with PBS+. Plates were cooled, and cells were scraped into tubes in a low volume of PBS+ 0.02% Triton X-100 and pelleted by centrifugation at 100g for 5 mins at 4°C before snap freezing in liquid nitrogen and storing at −80°C. Approximately 5 × 10^6^ cells were transferred to a Diagenode TPX tube and resuspended in ice-cold shearing buffer (1% Triton-X 100, 0.15 M NaCl, 1 mM EDTA, 0.5 mM EGTA, 20 mM HEPES/pH 7.6) containing 0.3% SDS and protease inhibitors. Chromatin was sheared using a Diagenode Bioruptor *Plus*: 20 cycles of 30sec on/30sec off on the high setting, and lysates were then diluted to 0.15% SDS and cleared by centrifugation at 14,000 RPM for 5 mins at 4°C. Then, 1/20 of the chromatin from ∼1 × 10^7^ cells was set aside and frozen for subsequent use as input control, and the remainder was incubated overnight at 4°C under rotation with 100μl of protein G dynabeads pre-loaded for 4 hrs at room temperature with 5μg of ChIP antibodies diluted in shearing buffer containing 0.15% SDS. Beads were magnetically immobilized, unbound supernatant was discarded and beads were sequentially washed under rotation twice with Wash Buffer 1 (0.1% SDS, 0.1% sodium deoxycholate, 1% Triton-X 100, 0.15 M NaCl, 1 mM EDTA, 0.5 mM EGTA, 20 mM HEPES/pH 7.6), once with Wash Buffer 2 (0.1% SDS, 0.1% sodium deoxycholate, 1% Triton-X 100, 0.5 M NaCl, 1 mM EDTA, 0.5 mM EGTA, 20 mM HEPES/pH 7.6), once with Wash Buffer 3 (0.5% sodium deoxycholate, 0.5% NP-40, 0.25 M LiCl, 1 mM EDTA, 0.5 mM EGTA, 20 mM HEPES/pH 7.6) and twice with Wash Buffer 4 (1 mM EDTA, 0.5 mM EGTA, 20 mM HEPES/pH 7.6) for 5 mins each, magnetically capturing beads between each wash. Chromatin was eluted from beads by incubating twice at 65°C for 10 mins in 100μl elution buffer (1% SDS, 0.1 M NaHCO_3_) on a shaking heat block, capturing beads between each elution step and then pooling each eluted fraction. Input samples were made up to 200μl with elution buffer, 6.4μl of 5 M NaCl was added to each input or immunoprecipitated sample, and all samples were de-crosslinked overnight at 65°C. Samples were incubated for 2 hrs at 37°C with 0.2μg/ml PureLink RNAse A, then supplemented with 5 mM EDTA and incubated for an additional 2 hrs at 45°C with 0.2μg/ml proteinase K before purifying DNA with Qiagen PCR clean-up columns.

Immunoprecipitated DNA was analysed by qPCR using the Applied Biosystems QuantStudio Real Time PCR system and analysed with Applied Biosystems QuantStudio 12K Flex software. PCR primers were designed using the online PrimerBLAST design tool and validated (standard curve and melting curve) or taken from previously published papers. Primer sequences are detailed in Table S1. Enrichment values for each region of interest were calculated by normalization against a no antibody control. Each ChIP analysis was repeated in at least in three independent experiments.

#### ChIP-seq, ATAC-seq and mRNA-seq data and processing

ATAC-seq data from day 2 epiblast-like (D2-EpiL), day 3 caudal epiblast-like (D3-CEpiL), day 4 (D4) and day 5 (D5) spinal cord (SC) were obtained from Metzis et al.^55^ (accession number E-MTAB-6337). Pseudo-bulk ATAC-seq generated from mouse embryo 10x multiome experiments were obtained from Argelaguet et al.^56^ (accession number GSE205117). ChIP-seq data from naïve mouse ESCs, caudal epiblast-like cells and *Sox2* over-expressing caudal-epiblast-like cells (Figures 3A and S4E) were obtained from Blassberg et al.^57^ (accession number GSE162774). ChIP-seq data against histone marks were obtained from Yang et al.^62^ (accession number GSE98101) for embryos and from Mazzoni et al.^33^ (accession number GSE39433) for differentiated mouse ESCs. ChIP-seq data against HDAC3 and p300 were respectively obtained from Zylicz et al.^61^ (accession number GSE116480) and Amin et al.^9^ (accession number GSE84899). ChIP-seq data against H3K27me3, SUZ12 and JARID2 in mESCs were obtained from Kanellopoulou et al.^63^ (accession number GSE60397). Big Wig tracks were visualised using IGV.^114^

ATAC-seq signals were overlapped with the regulatory elements: *lee, Intron1*, *Prom*, *5’*, and *Tee*. The overall signal was calculated as the mean signal within the region, and the mean signal was normalised to 1 over the intensity of all analysed regions. Relative fold change to the final condition (D5SC for *in vitro* samples and SC for *in vivo* samples) was calculated for each region.

For mRNA-seq data^31^ (accession number E-MTAB-2268), the nf-core/rnaseq pipeline (version 2.0)^111^ was used with default parameters. Briefly, the pipeline performs quality control, trimming (using TrimGalore!), (pseudo-)alignment (using Salmon), and produces a gene expression matrix. All data were processed relative to the mouse UCSC mm10 genome (UCSC) downloaded from AWS iGenomes (https://github.com/ewels/AWS-iGenomes).

#### Identification of Transcription Factor Binding Sites (TFBS)

To identify transcription factor binding sites (TFBS) in genomic sequences, we used a comprehensive bioinformatics approach with publicly available databases and specialised software tools. The matrices representing the binding preferences of transcription factors were obtained from the JASPAR2022 database.^116^

To detect TFBS instances within our specific sequences, we used TFBStools version 1.40.0, as described in Tan and Lenhard.^113^ We set TFBStools to search for matches to the JASPAR matrices within our sequences, specifying an identity match threshold of 80%. The threshold was selected to identify low-affinity sites for TFs of interest in our specified regions. Then, we filtered our list to remove any non-expressed transcription factors based on mRNA-seq data generated *in vitro* in EpiL, CEpiL, D4 SC and D5 SC.^31^

The conservation profiles of *P1* and *P2*, as well as the multiple sequence alignment of the RARB motif in *P2* were visualised in the UCSC genome browser. The tracks used are available in the following session: https://genome-euro.ucsc.edu/s/da_bar/cdx2_control_conservation.

#### Prediction of Protein-Protein interaction Complexes

To further investigate the functional implications of identified TFBS instances, we aimed to predict the structures of protein-protein complexes involving our TFs of interest. To do this, we retrieved the complete TF protein sequences from InterPro, a comprehensive database of protein families, domains, and functional sites.^117^ We then used ColabFold version 1.3.0, which is an interface for AlphaFold-multimer program.^118^ AlphaFold-multimer is a state-of-the-art method for predicting protein complex structures, leveraging deep learning to estimate the three-dimensional arrangements of protein subunits.^67^^,^^119^ By inputting the TF protein sequences into ColabFold, we were able to obtain high-confidence predictions of their potential interactions and complex formations.

#### Experimental design

No statistical method was used to pre-determine sample size. No data were excluded from the analyses. The experiments were not randomized. The investigators were not blinded to allocation during experiments and outcome assessment. For each experiment, data were obtained from a minimum of three independent experiments.

### Quantification and statistical analysis

For all statistical analyses, data were obtained from a minimum of three independent experiments. Technical and biological replicates were pooled. The number of biological and technical replicates are indicated in Table S4. Bars denote mean +/- s.e.m and statistical significance was calculated using GraphPad Prism (GraphPad Software). Each dot represents a replicate. Details of the statistical analyses performed for each experiment are specified in Table S4. No methods were used to determine whether the data followed a normal distribution.

## Supplemental information

Document S1. Figures S1–S4 and Tables S1, S2, and S4

Table S3. TF binding sites identified in *P1* versus *P2*, related to Figure 4

Document S2. Article plus supplemental information
