# Supplementary material for: A dual enhancer-attenuator element ensures transient Cdx2 expression during mouse posterior body formation
Source: Dev Cell. 2025 Sep 22;60(18):2407–2419.e6. doi: 10.1016/j.devcel.2025.06.006 (PMC12979250; doi:10.1016/j.devcel.2025.06.006)
Supplement: Document S1. Figures S1–S4 and Tables S1, S2, and S4 [file mmc1.pdf]

Developmental Cell, Volume *60*

Supplemental information

A dual enhancer-attenuator element ensures transient *Cdx2* expression during mouse posterior body formation

Irène Amblard, Damir Baranasic, Sheila Q. Xie, Benjamin Moyon, Michelle Percharde, Boris Lenhard, and Vicki Metzis

Figure S1

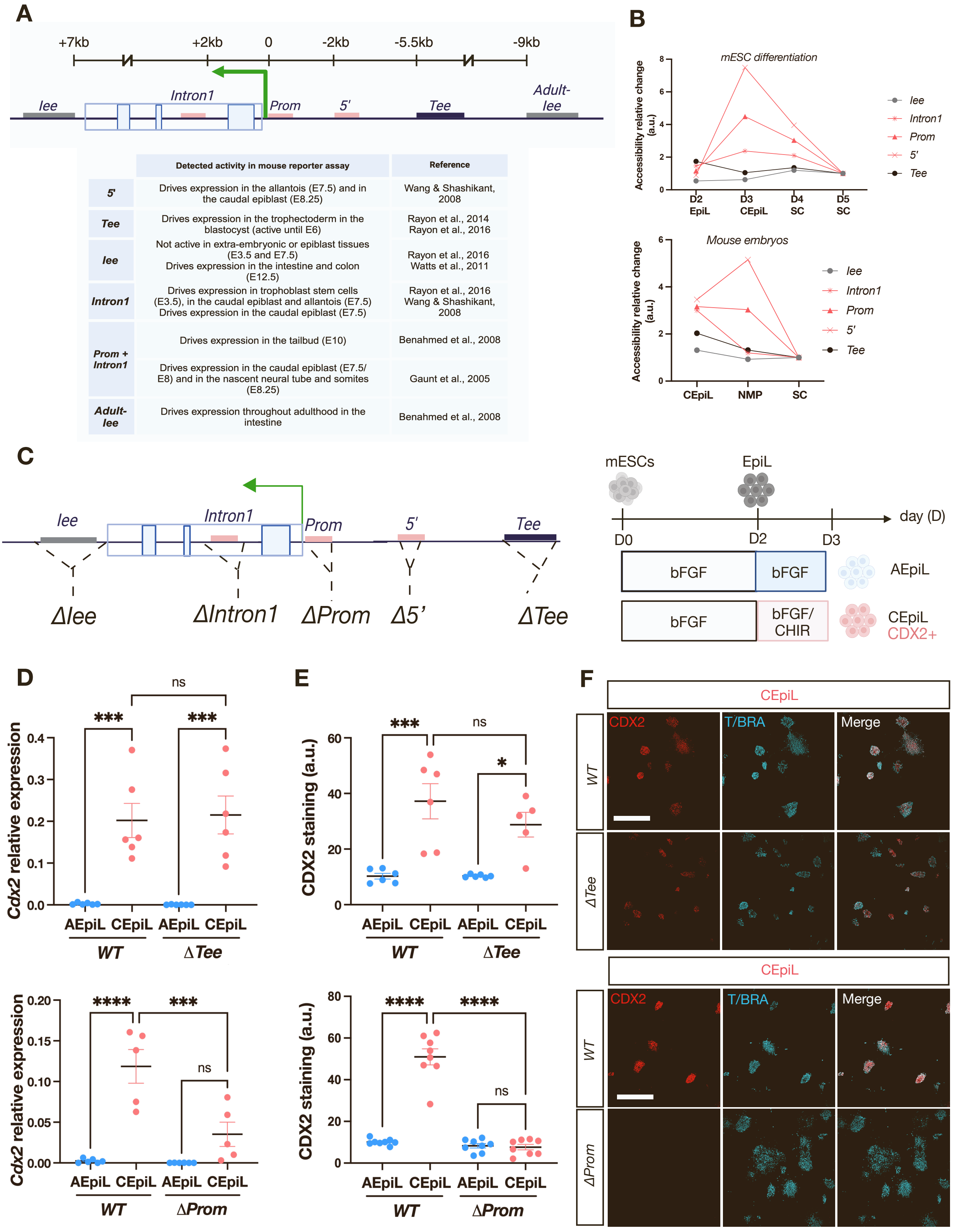

	Detected activity in mouse reporter assay	Reference
5'	Drives expression in the allantois (E7.5) and in the caudal epiblast (E8.25)	Wang & Shashikant, 2008
Tee	Drives expression in the trophectoderm in the blastocyst (active until E6)	Rayon et al., 2014 Rayon et al., 2016
Iee	Not active in extra-embryonic or epiblast tissues (E3.5 and E7.5) Drives expression in the intestine and colon (E12.5)	Rayon et al., 2016 Watts et al., 2011
Intron1	Drives expression in trophoblast stem cells (E3.5), in the caudal epiblast and allantois (E7.5) Drives expression in the caudal epiblast (E7.5)	Rayon et al., 2016 Wang & Shashikant, 2008
Prom + Intron1	Drives expression in the tailbud (E10)	Benahmed et al., 2008
	Drives expression in the caudal epiblast (E7.5/E8) and in the nascent neural tube and somites (E8.25)	Gaunt et al., 2005
Adult-Iee	Drives expression throughout adulthood in the intestine	Benahmed et al., 2008

Figure S1 *Cdx2* expression in caudal epiblast progenitors is not affected by the removal of the *Tee* element. Related to Figure 1.

(**A**) Simplified schematic of the proximally-located *Cdx2* cis-regulatory elements previously analysed by reporter assays and their distance to the promoter (not drawn to scale). (**B**) ATAC-seq quantification for each region of interest across different conditions. Fold change of each region is quantified relatively to day 5 spinal cord (mESC differentiation) or spinal cord (mouse embryos). (**C**) Schematic of the *Cdx2* locus illustrating the position of elements targeted for removal in mouse ESCs and conditions used to generate caudal epiblast-like cells (pink) versus control anterior epiblast-like cells (blue). (**D**) Relative expression (RT-qPCR) for *Cdx2* confirms that *Cdx2* is lost in CEpiL cells lacking the promoter (*ΔProm*) but not *Tee*-lacking cells (*ΔTee*). (**E-F**) CDX2 levels assessed by cytometry (E) and immunofluorescence (F) from *ΔTee*, and *ΔProm* cells show CDX2 is not induced in CEpiL cells that lack the promoter element while the removal of the *Tee* and has no effect on *Cdx2*. Scale bar represents 500μm. n=3. Panels A and C created with BioRender.com. Data are represented as mean ± SEM. AEpiL = anterior epiblast-like; CEpiL = caudal epiblast-like; EpiL = epiblast-like; PSM = presomitic mesoderm; SC = spinal cord.

Figure S2

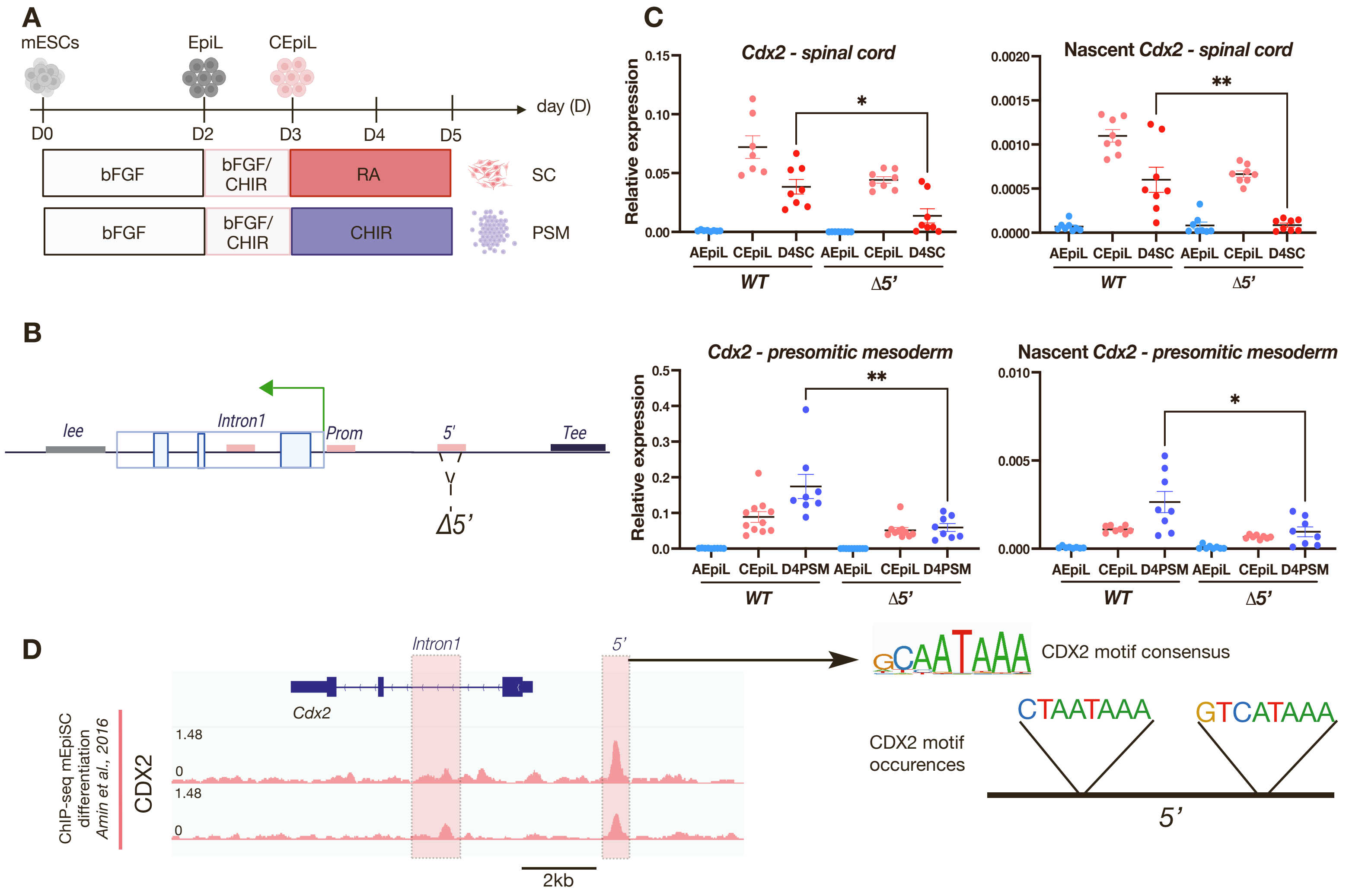
A
mESCs
EpiL
CEpiL
day (D)
D0
D2
D3
D4
D5
bFGF
bFGF/ CHIR
RA
SC
bFGF
bFGF/ CHIR
CHIR
PSM
B
Iee
Intron1
Prom
5'
Tee
Δ5'
C
Cdx2 - spinal cord
Nascent Cdx2 - spinal cord
Relative expression
AEpiL CEpiL D4SC AEpiL CEpiL D4SC
WT
Δ5'
Cdx2 - presomitic mesoderm
Nascent Cdx2 - presomitic mesoderm
AEpiL CEpiL D4PSM AEpiL CEpiL D4PSM
D
Intron1
5'
Cdx2
ChIP-seq mEpiSC differentiation Amin et al., 2016
CDX2
1.48
0
2kb
CDX2 motif consensus
CDX2 motif occurences
CTAATAAA
GTCATAAA
5'

Figure S2: Removal of the 5' element disrupts *Cdx2* nascent transcription. Related to Figure 2.

(**A**) Schematic of the conditions used to generate caudal epiblast-like cells from mouse ESCs for subsequent differentiation into spinal cord or presomitic mesoderm progenitors. (**B**) Schematic illustrating the deleted region in *Δ5'* cells. (**C**) RT-qPCR detecting *Cdx2* spliced and nascent *Cdx2* transcript shows a significant reduction in *Cdx2* in Δ*5*' day 4 SC and day 4 PSM cells versus WT day 4 SC and day 4 PSM cells. (**D**) ChIP-seq signal for CDX2 in caudal epiblast-like conditions from the indicated study reveals occupancy at the *5'* element, which contains two motifs for CDX2. Panels A-B created with BioRender.com. Data are represented as mean ± SEM. AEpiL = anterior epiblast-like; CEpiL = caudal epiblast-like; EpiL = epiblast-like; PSM = presomitic mesoderm; SC = spinal cord.

Figure S3

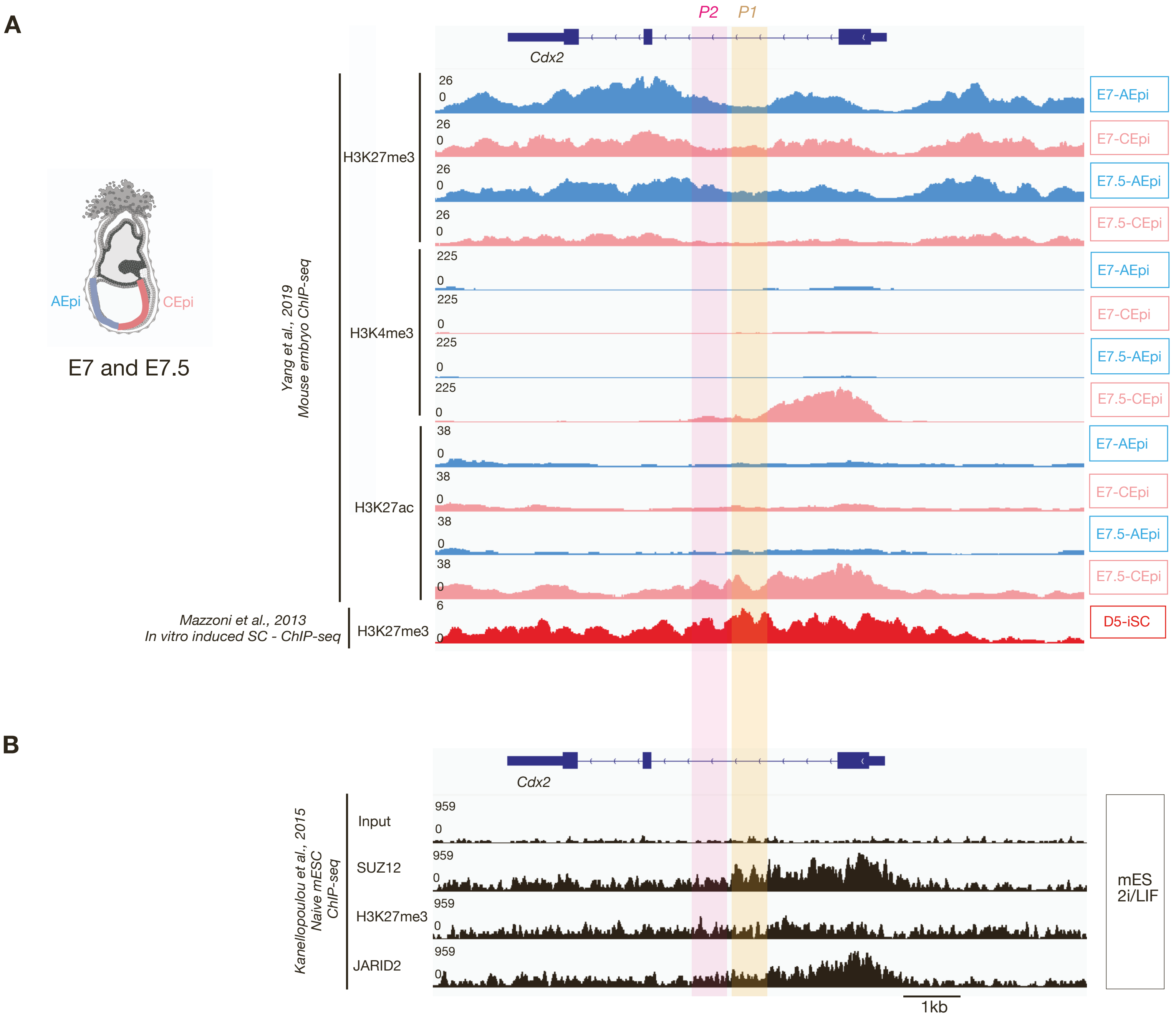

A
P2
P1
Cdx2
E7 and E7.5
AEpi
CEpi
Yang et al., 2019
Mouse embryo ChIP-seq
H3K27me3
H3K4me3
H3K27ac
E7-AEpi
E7-CEpi
E7.5-AEpi
E7.5-CEpi
26
225
38
0
Mazzoni et al., 2013
In vitro induced SC - ChIP-seq
H3K27me3
6
D5-iSC
B
Cdx2
Kanellopoulou et al., 2015
Naive mESC
ChIP-seq
Input
SUZ12
H3K27me3
JARID2
959
mES
2i/LIF
1kb

Figure S3: Histones marks are broadly detected at *Cdx2*. Relates to Figure 3.

(**A**) ChIP-seq signal for H3K27me3, H3K4me3, H3K27ac in sub-dissected anterior (blue) or caudal (pink) epiblast tissues from E7 and E7.5 embryos and H3K27me3 from mouse ESC-derived spinal cord progenitors from the indicated studies demonstrates a broad signal detected around the gene body of *Cdx2* both *in vivo* and *in vitro*. Embryo schematic created with BioRender.com. (**B**) The ChIP-seq signal for H3K27me3, SUZ12 and JARID2 in naive mouse ESCs from the indicated study shows a similar distribution.

Figure S4

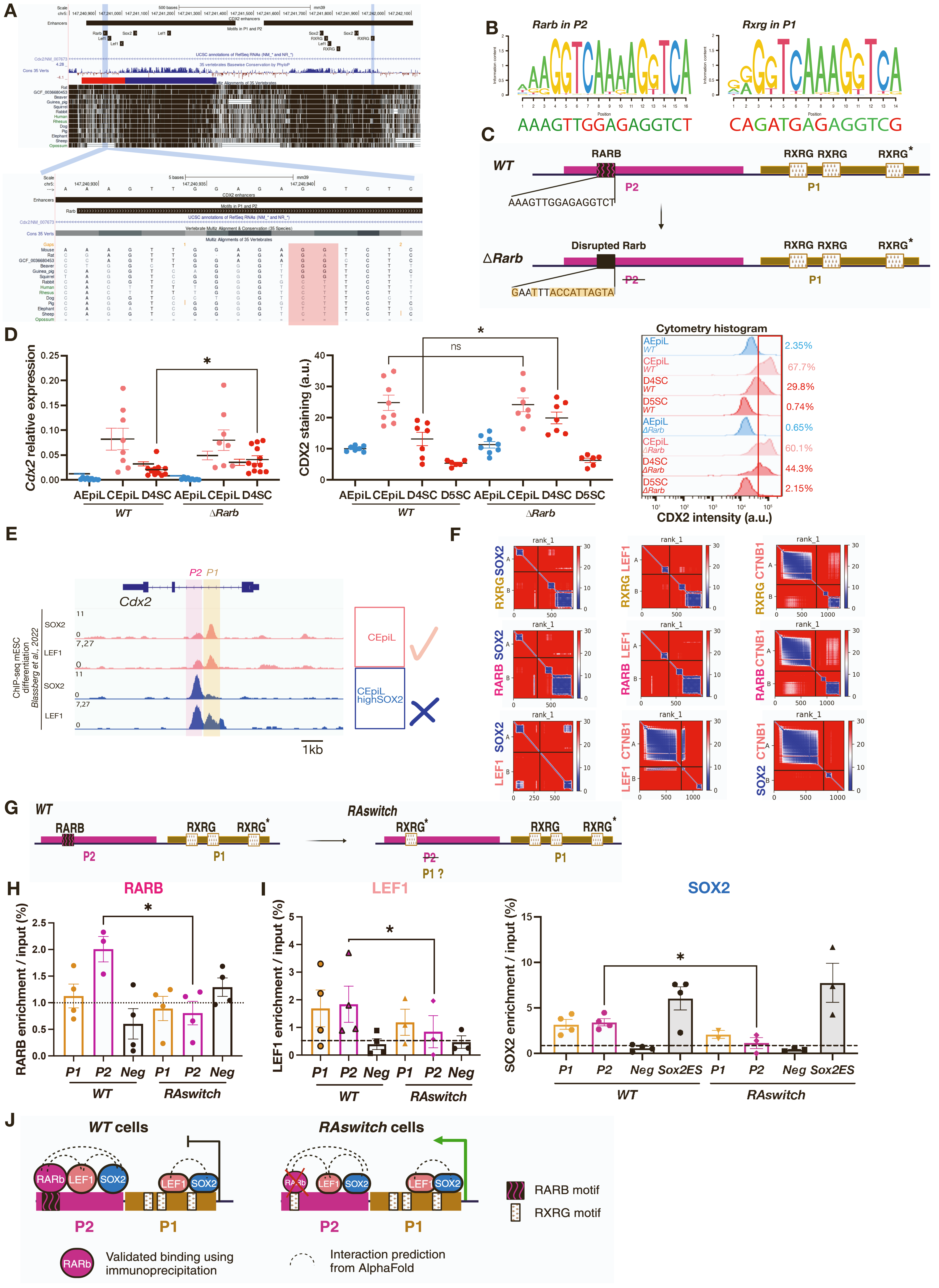
A
B
Rarb in P2
Rxrg in P1
AAAGTTGGAGAGGTCT
CAGATGAGAGGTCG
C
WT
RARB
RXRG RXRG
RXRG*
P2
P1
AAAGTTGGAGAGGTCT
ΔRarb
Disrupted Rarb
GAATTTACCATTAGTA
D
Cdx2 relative expression
CDX2 staining (a.u.)
AEpiL CEpiL D4SC AEpiL CEpiL D4SC
AEpiL CEpiL D4SC D5SC AEpiL CEpiL D4SC D5SC
WT
ΔRarb
ns
Cytometry histogram
2.35%
67.7%
29.8%
0.74%
0.65%
60.1%
44.3%
2.15%
CDX2 intensity (a.u.)
E
Cdx2
ChIP-seq mESC differentiation Blassberg et al., 2022
SOX2
LEF1
CEpiL
CEpiL highSOX2
1kb
F
rank_1
RXRG SOX2
RXRG LEF1
RXRG CTNB1
RARB SOX2
RARB LEF1
RARB CTNB1
LEF1 SOX2
LEF1 CTNB1
SOX2 CTNB1
G
WT
RAswitch
P1 ?
H
RARB
RARB enrichment / input (%)
P1 P2 Neg P1 P2 Neg
I
LEF1
LEF1 enrichment / input (%)
SOX2
SOX2 enrichment / input (%)
P1 P2 Neg Sox2ES P1 P2 Neg Sox2ES
J
WT cells
RAswitch cells
RARb LEF1 SOX2
RARB motif
RXRG motif
Validated binding using immunoprecipitation
Interaction prediction from AlphaFold

Figure S4: RA and WNT signalling effectors interact *in silico*. Relates to Figure 4.

(**A**) UCSC Genome Browser view of *P1* and *P2* regions including transcription factor binding sites (TFBS), gene model, and phyloP conservation scores (positive: conservation; negative: acceleration). Highlighted in *P2* are an evolutionary accelerated domain (red) and a constrained domain (blue), alongside a multiple sequence alignment. The bottom panel shows a zoom-in on the RARB binding site in *P2*, displaying the base-pair resolution of the alignment**.** (**B**) Alignment between RARB and RXRG TFBS extracted from JASPAR with their respective matches in *P2* and *P1*. The motif is represented as the sequence logo, showing the nucleotide preference of the motif at each position. Green and red letters in the genomic TFBS sequence represent matches and mismatches with the motif consensus sequence, respectively. (**C**) Schematic illustrating the mutated basepairs (yellow) within the RARB motif in *ΔRarb* cells. (**D**) RT-qPCR and flow cytometry shows an increase in *Cdx2* spliced transcript and CDX2 protein in *ΔRarb* day 4 SC cells. (**E**) ChIP-seq signals at the *Cdx2* locus showing that in conditions that repress CDX2 by inducing high levels of SOX2 in caudal epiblast cells, SOX2 and LEF1 are detected at *P2* from the indicated study. (**F**) Protein contact probability matrices calculated by AlphaFold-Multimer show that RARB and RXRG interact with LEF1 and SOX2 *in silico*. Lower values represent higher contact probabilities. Protein sequences are divided by the black line. The upper right and lower left quadrants represent predicted protein-protein interactions. (**G**) Schematic showing that *RAswitch* ESCs contain a single copy of the RXRG motif from *P1* (indicated with an asterix) introduced at *P2* to substitute for the single copy of RARB present in WT cells. (**H**) RARB enrichment, comparing *RAswitch* to WT day 4 SC cells, at *P1* and *P2.* The negative control region (neg) represents an RARE-negative region from Kumar & Duester, 2014 [S1]. (**I**) LEF1 and SOX2 enrichment comparing *RAswitch* to *WT* day 4 SC cells at *P1* and *P2* using a non-bound region as a negative control (Neg) and Sox2ES as a SOX2-bound region (**J**) LEF1 and SOX2 accumulate at *P2* in day 4 SC in a RARB-dependent manner. Panels C,G and J created with BioRender.com. Data are represented as mean ± SEM. AEpiL = anterior epiblast-like; CEpiL = caudal epiblast-like; EpiL = epiblast-like; PSM = presomitic mesoderm; SC = spinal cord.

Figure S4: RA and WNT signalling effectors interact *in silico*. Relates to Figure 4.

(**A**) UCSC Genome Browser view of *P1* and *P2* regions including transcription factor binding sites (TFBS), gene model, and phyloP conservation scores (positive: conservation; negative: acceleration). Highlighted in *P2* are an evolutionary accelerated domain (red) and a constrained domain (blue), alongside a multiple sequence alignment. The bottom panel shows a zoom-in on the RARB binding site in *P2*, displaying the base-pair resolution of the alignment**.** (**B**) Alignment between RARB and RXRG TFBS extracted from JASPAR with their respective matches in *P2* and *P1*. The motif is represented as the sequence logo, showing the nucleotide preference of the motif at each position. Green and red letters in the genomic TFBS sequence represent matches and mismatches with the motif consensus sequence, respectively. (**C**) Schematic illustrating the mutated basepairs (yellow) within the RARB motif in *ΔRarb* cells. (**D**) RT-qPCR and flow cytometry shows an increase in *Cdx2* spliced transcript and CDX2 protein in *ΔRarb* day 4 SC cells. (**E**) ChIP-seq signals at the *Cdx2* locus showing that in conditions that repress CDX2 by inducing high levels of SOX2 in caudal epiblast cells, SOX2 and LEF1 are detected at *P2* from the indicated study. (**F**) Protein contact probability matrices calculated by AlphaFold-Multimer show that RARB and RXRG interact with LEF1 and SOX2 *in silico*. Lower values represent higher contact probabilities. Protein sequences are divided by the black line. The upper right and lower left quadrants represent predicted protein-protein interactions. (**G**) Schematic showing that *RAswitch* ESCs contain a single copy of the RXRG motif from *P1* (indicated with an asterix) introduced at *P2* to substitute for the single copy of RARB present in WT cells. (**H**) RARB enrichment comparing *RAswitch* to WT day 4 SC cells at *P1* and *P2* using a RARE-negative control from Kumar & Duester, 2014 [S1] as a RARE-negative control. (**I**) LEF1 and SOX2 enrichment comparing *RAswitch* to *WT* day 4 SC cells at *P1* and *P2* using a non-bound region as a negative control (Neg) and Sox2ES as a SOX2-bound region (**J**) LEF1 and SOX2 accumulate at *P2* in day 4 SC in a RARB-dependent manner. Panels C,G and J created with BioRender.com. Data are represented as mean ± SEM. AEpiL = anterior epiblast-like; CEpiL = caudal epiblast-like; EpiL = epiblast-like; PSM = presomitic mesoderm; SC = spinal cord.

Table S1: Primers used for qPCR, related to STAR Methods
Table S2: sgRNA sequence, genotyping and sequencing primers *in vitro* and *in vivo,* related to STAR Methods
Table S3: Transcription factor binding sites identified in P1 vs P2, related to Figure 4

Table S1: Primers used for qPCR, related to STAR Methods

Name	Sequence (5-->3)	Reference
Actin_FOR	TGGCTCCTAGCACCATGA	Metzis et al. [S2]
Actin_REV	CCACCGATCCACACAGAG	
Cdx2_FOR	TAGTCGATACATCACCATCAGG	
Cdx2_REV	TGATTTTCCTCTCCTTGGCTCT	
NascentCdx2_FOR	TGCCCCAGGCCATAATAACC	This paper
NascentCdx2_REV	GAGGAAAAGTGAGCTGGCTG	
Rarb_FOR	GAGAACAGTGGAGTCAGTCAGTC	Shibata et al. [S3]
Rarb_REV	GCTGGAAATGTCTCACTGCA	
P2_FOR	GGGACCTCTCTCTGATCCCT	This paper
P2_REV	CGGGGATGCTTCAATGGACA	
P1_FOR	GGGTGGTGACTTTCGCAGT	
P1_REV	CGCCCTAGAACGGATGCTG	
Neg_FOR (RARB)	TAGCAGCTGAATGAGTGGCTCTA	Kumar et al. [S1]
Neg_REV (RARB)	GTAGCAAGCAGTTACCTGATCTG	
Neg_FOR (LEF1/SOX2)	CAGAGGACAGCTAGGCAGAGA	Kujetova et al. [S4]
Neg_REV (LEF1/SOX2)	GAGCAGACTCATTAGGGCAAA	
Sox2ES_FOR	GGCTCGGGCAGCCATT	
Sox2ES_REV	ACTGTCGACTGTGCTCATTACCA	

Table S2: sgRNA sequence, genotyping and sequencing primers used for *in vitro* and *in vivo* targeting, related to STAR Methods

Cell line	sgRNA sequence (5-->3)	Genotyping primer	Sequencing primer
ΔIee	Upstream – GGCAAAAAGCTCGTCACAA	CAGCGTGTATGTGTGCCAGTGTAC	CAGCGTGTATGTGTGCCA GTGTAC
	Downstream- CAGTGTACCCCGTCTGAGG	TGGAGGTGATGGGGTAGAGAAGG	
Δ5'	Upstream- GCTTGGGCACCCAACCGCT	GCCCAGGTTTTGCAAGTCAG	AGGTACCTTGCAGGAACA GCAAG
	Downstream- GCCGGCATTCGGAAGACAC	AGGTACCTTGCAGGAACAGCAAG	
ΔTee	Upstream- TAAATGACTTCCCCAAC	GTTAAGGGCTTCTCTTGGCA	TTCTGTGACTCAACTTGG AAAGC
	Downstream- CGGCGGGCAATGCGCGC	TTCTGTGACTCAACTTGGAAAGC	
ΔP1	Upstream- AGTCGGGCTCCGGCACTG	TGCCTGAAAGGTAGTGAGCG	GACCTTGACAAATAGCGC GG
	Downstream- GGCGCCTGCAAGCCCTTG	GACCTTGACAAATAGCGCGG	
ΔP2	Upstream- AGCCCGCTCCCGACCGGT	TGCCTGAAAGGTAGTGAGCG	GACCTTGACAAATAGCGC GG
	Downstream- ATTCAACCCGGTTAAGTCG	GACCTTGACAAATAGCGCGG	
ΔProm	Upstream- CTAATTACACGACGTATT	TAGGTGTCAGCACAAATACTTCCC	AGGTAGCTCACGTACATG GTG
	Downstream- GAGCCGACGGAGCACCGT	ACCATTGAGACCGTGGGCTA	
ΔIntron 1	Upstream- AGTCGGGCTCCGGCACTG	TGCCTGAAAGGTAGTGAGCG	GACCTTGACAAATAGCGC GG
	Downstream- ATTCAACCCGGTTAAGTCG	GACCTTGACAAATAGCGCGG	
ΔRarb	sgRNA – TGTTCCACGTCCAAAGTG	GGCAACTCCCACCCCTTAGA	GTCCCGACCTCTCATCTG C
		GTTCCACGTCCCGACCTCTC	
RA-switch	sgRNA – TGTTCCACGTCCAAAGTG Repair oligo- TTCAATGGACAGAATGCTG GCCAGGAACTGTTCCACGTC CCGACCTCTCATCTGCTCACATC AAAACAGATAGATTTTAGTGCTC AGTGCCTAGTTG	GGCAACTCCCACCCCTTAGA	GTCCCGACCTCTCATCTG C
		GTTCCACGTCCCGACCTCTC	
ΔP2 in vivo	Upstream - AGCCCGCTCCCGACCGGT<br>Downstream - ATTCAACCCGGTTAAGTCG	TGCCTGAAAGGTAGTGAGCG<br>GACCTTGACAAATAGCGCGG	GACCTTGACAAATAGCGC GG
ΔRarb in vivo	TGTTCCACGTCCAAAGTG	TGCCTGAAAGGTAGTGAGCG<br>GACCTTGACAAATAGCGCGG	GACCTTGACAAATAGCGC GG

Table S4: Statistics across all datasets, related to Figure 1-4

Fig	Cell line	Condition	n	p-value	p-value
1E-Intron1	WT	AEpiL	6	p<0.0001	WT vs *ΔIntron1* CEpiL<br>p<0.0001<br>Tukey's t-test
		CEpiL	8		
	ΔIntron1	AEpiL	6	p=0.5487	
		CEpiL	8		
1E-5'	WT	AEpiL	7	p<0.0001	WT vs *Δ5'* CEpiL<br>p=0.0436<br>Tukey's t-test
		CEpiL	8		
	Δ5'	AEpiL	8	p=0.0001	
		CEpiL	8		
1E-lee	WT	AEpiL	5	p=0.0345	WT vs *Δlee* CEpiL<br>p=0.5605<br>Tukey's t-test
		CEpiL	6		
	Δlee	AEpiL	5	p=0.0048	
		CEpiL	5		
1F-Intron1	WT	AEpiL	10	p<0.0001	WT vs *ΔIntron1* CEpiL<br>p<0.0001<br>Tukey's t-test
		CEpiL	10		
	ΔIntron1	AEpiL	10	p=0.1269	
		CEpiL	10		
1F-5'	WT	AEpiL	8	p=0.0001	WT vs *Δ5'* CEpiL<br>p=0.3495<br>Tukey's t-test
		CEpiL	8		
	Δ5'	AEpiL	8	p=0.0067	
		CEpiL	8		
1F-lee	WT	AEpiL	6	p=0.0025	WT vs *Δlee* CEpiL<br>p=0.9990<br>Tukey's t-test
		CEpiL	6		
	Δlee	AEpiL	5	p=0.0094	
		CEpiL	5		
2D	WT	CEpiL	6		WT vs *ΔIntron1* CEpiL<br>p<0.0001<br>WT vs *ΔIntron1* D4PSM<br>p= 0.2349 t-test
		D4PSM	8		
	ΔIntron1	CEpiL	6		
		D4PSM	8		
2F-SC	WT	D4SC	5		WT vs *Δ5'* D4SC<br>p=0.0281; two-tailed t-test
	Δ5'	D4SC	5		
2F-PSM	WT	D4PSM	6		WT vs *Δ5'* D4PSM<br>p=0.0015<br>WT vs *Δ5'* D5PSM p=0.0883<br>t-test
		D5PSM	6		
	Δ5'	D4PSM	6		
		D5PSM	6		
3C	WT	AEpiL	13		WT vs *ΔP1* CEpiL<br>p=0.0017<br>WT vs *ΔP2* CEpiL<br>p=0.1592<br>*ΔP1* vs *ΔP2* CEpiL p<0.0001<br>t-test
		CEpiL	13		
	ΔP1	AEpiL	6		
		CEpiL	6		
	ΔP2	AEpiL	8		
		CEpiL	8		
3E-spliced	WT	CEpiL	10	WT CEpiL vs D4SC p=0.0343<br>t-test	WT vs *ΔP2* D4SC p=0.0283<br>t-test
		D3.5SC	14		
		D4SC	14		
	ΔP2	CEpiL	10	*ΔP2* CEpiL vs D4SC p=0.2753<br>t-test	
		D3.5SC	15		
		D4SC	14		
3E-nascent	WT	CEpiL	10	WT CEpiL vs D3.5SC p=0.0103<br>t-test	WT vs *ΔP2* D3.5SC p=<br>0.0453<br>WT vs *ΔP2* D4SC p=0.2325<br>t-test
		D3.5SC	17		
		D4SC	17		
	ΔP2	CEpiL	10	*ΔP2* CEpiL vs D3.5SC p=0.1862<br>*ΔP2* CEpiL vs D4SC p=0.0291 t-test	
		D3.5SC	17		
		D4SC	17		
3F	WT	D4SC	5		WT vs *ΔP2* D4SC<br>p=0.0333, t-test
	ΔP2	4SC	6		

4C-Rarb	WT	D4SC 0	7	D4SC 0 vs D4SC 100 p=0.9538 D4SC 0 vs D4SC 1000 p<0.0001 t-test	
		D4SC 100	8		
		D4SC1000	11		
4C-Cdx2	WT	D4SC 0	7	D4SC 0 vs D4SC 100 p=0.0388 D4SC 0 vs D4SC 1000 p= 0.0160 t-test	
		D4SC 100	8		
		D4SC1000	11		
4E-FACS	WT	CEpiL	6		WT vs RAswitch CEpiL p=0.0163 WT vs RAswitch D4SC p=0.0212 WT vs RAswitch D5SC p=0.0068 t-test
		D4SC	10		
		D5SC	8		
	RAswitch	CEpiL	7		
		D4SC	10		
		D5SC	8		
4E-spliced	WT	CEpiL	8		WT vs RAswitch CEpiL p=0.0035 WT vs RAswitch D4SC p= 0.0582 t-test
		D4SC	10		
	RAswitch	CEpiL	8		
		D4SC	10		
4E-nascent	WT	CEpiL	8		WT vs RAswitch CEpiL p=0.0452 WT vs RAswitch D4SC p= 0.1064 t-test
		D4SC	8		
	RAswitch	CEpiL	8		
		D4SC	10		
S1D-Tee	WT	AEpiL	6	p=0.0009	WT vs *ΔTee* CEpiL p=0.9898 Tukey's t-test
		CEpiL	6		
	ΔTee	AEpiL	6	p=0.0004	
		CEpiL	6		
S1D-Prom	WT	AEpiL	5	p<0.0001	WT vs *ΔProm* CEpiL p=0.0006 Tukey's t-test
		CEpiL	5		
	ΔProm	AEpiL	5	p=0.1761	
		CEpiL	5		
S1E-Tee	WT	AEpiL	6	p=0.0004	WT vs *ΔTee* CEpiL p=0.4537 Tukey's t-test
		CEpiL	6		
	ΔTee	AEpiL	6	p=0.0185	
		CEpiL	5		
S1E-Prom	WT	AEpiL	8	p<0.0001	WT vs *ΔProm* CEpiL p<0.0001 Tukey's t-test
		CEpiL	8		
	ΔProm	AEpiL	8	p=0.9961	
		CEpiL	8		
S2C-spliced-SC	WT	D4SC	8		WT vs *Δ5'* D4SC p=0.0129 t-test
	Δ5'	D4SC	8		
S2C-nascent-spliced	WT	D4SC	8		WT vs *Δ5'* D4SC p=0.0031 t-test
	Δ5'	D4SC	8		
S2C-spliced-PSM	WT	D4PSM	8		WT vs *Δ5'* D4PSM p=0.006 t-test
	Δ5'	D4PSM	8		
S2C-nascent-PSM	WT	D4PSM	8		WT vs *Δ5'* D4PSM p=0.0233 t-test
	Δ5'	D4PSM	8		
S4D-qPCR	*WT*	D4SC	11		WT vs *ΔRarb* D4SC p=0.0262 t-test
	ΔRarb	D4SC	12		
S4D-FACS	*WT*	D4SC	7		WT vs *ΔRarb* D4SC p=0.0361 t-test
	ΔRarb	D4SC	7		
S4H	*WT*	P2	3		WT vs RAswitch P2 p=0.0147 t-test
	RAswitch	P2	4		
S4I-LEF1	*WT*	P2	4		WT vs RAswitch P2 p= 0.0300 t-test
	RAswitch	P2	3		
S4I-SOX2	*WT*	P2	4		WT vs RAswitch P2 p= 0.0199 t-test
	RAswitch	P2	3		

Supplemental References

S1.
Kumar, S., and Duester, G. (2014). Retinoic acid controls body axis extension by directly repressing Fgf8 transcription. Development 141, 2972–2977. https://doi.org/10.1242/dev.112367

S2.
Metzis, V., Steinhauser, S., Pakanavicius, E., Gouti, M., Stamataki, D., Ivanovitch, K., Watson, T., Rayon, T., Mousavy Gharavy, S.N., Lovell-Badge, R., et al. (2018). Nervous System Regionalization Entails Axial Allocation before Neural Differentiation. Cell 175, 1105-1118.e17. https://doi.org/10.1016/j.cell.2018.09.040

S3.
Shibata, M., Pattabiraman, K., Lorente-Galdos, B. et al. (2021). Regulation of prefrontal patterning and connectivity by retinoic acid. Nature 598, 483–488. https://doi.org/10.1038/s41586-021-03953-x

S4.
Kutejova, E., Sasai, N., Shah, A., Gouti, M., and Briscoe, J. (2016). Neural Progenitors Adopt Specific Identities by Directly Repressing All Alternative Progenitor Transcriptional Programs. Developmental Cell 36, 639–653. https://doi.org/10.1016/j.devcel.2016.02.013
